# Ultrasound‐Assisted H_2_ Transmitter Enables Hydrogen‐Gene Therapy to Prevent Anesthesia/Surgery‐Induced Cognitive Impairment

**DOI:** 10.1002/advs.202414397

**Published:** 2025-03-06

**Authors:** Ruonan Zhan, Yan Fang, Chuyun Lou, Nan Chen, Xuan Mo, Bo Jiao, Mengke Liu, Yangxi Zhao, Weichen Xu, Huixiong Xu, Haohao Yin, Yi Zhang

**Affiliations:** ^1^ Department of Anesthesiology and Pain Medicine Hubei Key Laboratory of Geriatric Anesthesia and Perioperative Brain Health, and Wuhan Clinical Research Center for Geriatric Anesthesia Tongji Hospital Tongji Medical College Huazhong University of Science and Technology Wuhan 430030 P. R. China; ^2^ Department of Ultrasound Institute of Ultrasound in Medicine and Engineering Zhongshan Hospital Fudan University Shanghai 200032 P. R. China; ^3^ Department of Ultrasound, Zhongshan Hospital (Xiamen) Fudan University Xiamen 361015 P. R. China; ^4^ Department of Ultrasound, Huashan Hospital Fudan University Shanghai 200040 China; ^5^ Department of Radiology The First Affiliated Hospital of Zhengzhou University No.1, Eastern Jianshe Road Zhengzhou Henan Province 450052 China; ^6^ Department of Medical Ultrasound Center of Minimally Invasive Treatment for Tumor Shanghai Tenth People's Hospital School of Medicine Tongji University Shanghai 200072 China

**Keywords:** focused ultrasound, hydrogen‐gene therapy, neuroinflammation, postoperative cognitive dysfunction (POCD), siRNA delivery

## Abstract

Postoperative cognitive dysfunction (POCD) frequently occurs after surgery, resulting in extended hospitalizations, higher healthcare expenses, and potential long‐term cognitive impairment. Despite its significant impact, effective preventive and therapeutic strategies for POCD are still lacking. Neuroinflammation plays a crucial role in the pathogenesis of POCD. To address this, a H_2_ emitter is developed that employs hydrogen‐gene therapy facilitated by ultrasound, allowing for the repair of the neuroinflammatory microenvironment in a spatiotemporally controllable manner to effectively prevent anesthesia/surgery‐induced cognitive impairment. Utilizing focused ultrasound, the blood–brain barrier can be opened in a controlled manner, enabling the efficient delivery of hydrogen emitters (HPPS) carrying siRNA to the site of neuroinflammation. On one hand, the hydrogen emitter effectively generates hydrogen to eliminate excess hydroxyl radicals; on the other hand, it utilizes siRNA to target and reduce tau protein phosphorylation. This targeted hydrogen‐gene therapy strategy has been demonstrated in both mouse and rat postoperative models to significantly reduce neuroinflammation and improve postoperative spatial memory as well as object recognition. This study introduces a novel and effective strategy for preventing anesthesia/surgery‐induced cognitive impairment and offering new insights for the treatment of other neuroinflammatory diseases.

## Graphical abstract

Postoperative cognitive dysfunction (POCD) remains a challenge with no effective treatments. This study introduces a novel hydrogen‐gene therapy strategy, combining focused ultrasound and siRNA delivery via hydrogen emitters, to repair neuroinflammation in a precise and controlled manner. Demonstrated in rodent models, this approach reduces neuroinflammation, improves cognitive function, and offers insights into treating neuroinflammatory diseases.

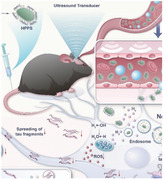

## 1 Introduction

Postoperative cognitive dysfunction (POCD) is marked by a decline in memory abilities, difficulties in maintaining attention, and problems with language skills, which generally arise following anesthesia and surgical procedures.^[^ ^1^ ^]^ The incidence of cognitive impairment within one‐week post‐surgery varies between 11% and 51%, depending on the type of surgery.^[^ ^2^, ^3^ ^]^ This syndrome frequently results in extended hospitalizations, rising healthcare expenses, elevated mortality rates, delayed recovery, and long‐term cognitive decline^[^ ^2^ ^]^ becoming a catalyst for more severe cognitive deterioration.^[^ ^4^ ^]^ Despite its impact, effective preventive measures for POCD remain elusive. Increasing evidence from our lab^[^ ^5^ ^]^ and others^[^ ^6^, ^7^ ^]^ suggests that neuroinflammatory processes are pivotal in the onset and development of POCD. Surgery‐induced stress activates microglia into a pro‐inflammatory state, contributing to a neuroinflammatory environment.^[^ ^8^, ^9^ ^]^ Increased phosphorylation of tau proteins has been observed in studies, which may further contribute to cognitive dysfunction.^[^ ^10^, ^11^ ^]^ POCD shares similarities in mechanisms and pathophysiology with Alzheimer's disease, where tau protein interactions with neuroinflammation are well‐documented. Over‐phosphorylated tau proteins can lead to axonal defects and neurofibrillary tangles, activating microglia and sustaining inflammation.^[^ ^12^ ^]^ This process creates a feedback loop that exacerbates neuronal damage, propagating tau protein spread and neuroinflammation.^[^ ^13^, ^14^, ^15^ ^]^ The stressors contributing to POCD may also involve endogenous LPS stimuli and pathological protein aggregates like tau. These can trigger microglial imbalances, releasing numerous immune factors that drive disease progression.^[^ ^16^ ^]^ Given that tau protein and neuroinflammation significantly contribute to the development of cognitive dysfunction, targeting the alleviation of neuroinflammation and the prevention of tau protein phosphorylation is expected to play a significant role in both the prevention and treatment of POCD.

Hydrogen therapy represents a potent anti‐inflammatory strategy. H₂ specifically targets and neutralizes highly toxic free radicals, like hydroxyl radicals (·OH), effectively reducing oxidative stress.^[^ ^17^ ^]^ This therapeutic approach has demonstrated significant efficacy in conditions such as myocardial ischemia‐reperfusion, atherosclerosis, and Alzheimer's disease.^[^ ^18^ ^]^ However, the uncontrolled distribution and low solubility of H₂ within biological systems limit its ability to accumulate at specific targets, thus constraining its therapeutic potential. Achieving a sufficiently high hydrogen concentration and ensuring prolonged exposure are critical for effective treatment. Thus, devising strategies for targeted, high‐capacity delivery and controlled release of hydrogen is crucial for improving therapeutic effects. Recent advancements in hydrogen‐generating nanoplatforms have facilitated the localized increase of hydrogen concentration in pathological tissues, offering substantial clinical potential.^[^ ^19^, ^20^ ^]^ Notably, SiH, as an efficient hydrogen‐releasing agent,^[^ ^21^ ^]^ features an intrinsic two‐dimensional structure with an ultra‐high surface area, providing abundant adsorption sites. This characteristic facilitates the simultaneous storage of hydrogen and the loading of tau‐siRNA, which may contribute to alleviating neuroinflammation in hippocampal tissues. Neuroinflammation is a key pathological feature in POCD, and tau hyperphosphorylation leads to abnormal aggregates linked to cognitive decline, making it a crucial therapeutic target. siRNA‐based therapies can specifically target tau, offering promise in precision medicine. siRNA therapy has gained attention as a potential treatment approach because of its capability to precisely silence genes associated with diseases.^[^ ^22^ ^]^ It offers a significant advantage in targeting previously “undruggable” genetic pathways. However, effective delivery remains a major challenge. The primary challenges in siRNA delivery include ensuring its stability in the bloodstream, as siRNA is prone to degradation by nucleases. Additionally, efficient cellular uptake is difficult due to its large molecular size and negative charge, which hinder membrane permeability. Targeted delivery to specific tissues or cells remains another major hurdle, as off‐target effects and immune activation can reduce therapeutic efficacy and increase side effects.

Nanomaterials, acting as carriers for siRNA, have transformed its delivery system by altering its susceptibility to degradation and overcoming the challenges posed by its negative charge and other unfavorable physical properties. This approach represents a promising strategy for siRNA delivery. SiH, a two‐dimensional nanosheet, can act as an siRNA carrier while generating hydrogen. The surface modification of SiH confers it with the capacity to protect siRNA, positioning it as a crucial component in hydrogen‐based gene therapy. This modification enables the possibility of dual‐targeted therapeutic strategies. Transporting siRNA to the brain presents significant difficulties, primarily due to the restrictive nature of the blood‐brain barrier (BBB).^[^ ^23^ ^]^ Microbubble‐assisted focused ultrasound (FUS+MBs) presents a low‐invasive strategy for temporarily disrupting the BBB.^[^ ^24^ ^]^ This approach increases local drug concentration and efficacy while reducing dosage and side effects. It is currently in preclinical trials.^[^ ^25^ ^]^

**Figure 1:**
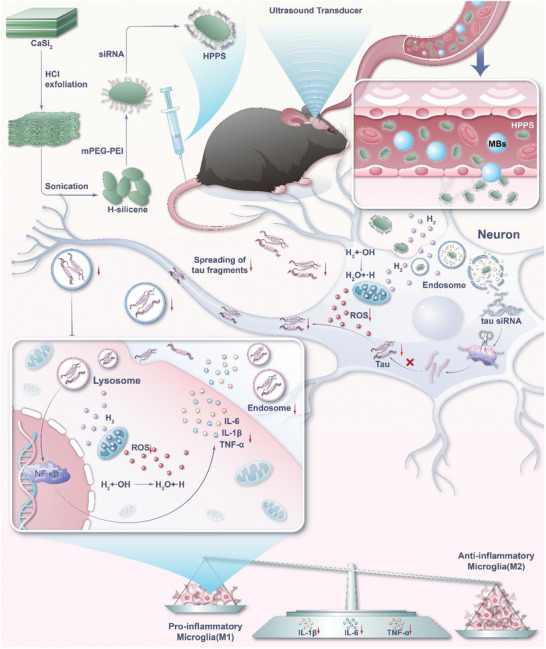
Schematic of the ultrasound‐assisted H₂ transmitter enabling hydrogen‐gene therapy for the prevention of anesthesia/surgery‐induced cognitive impairment.

In this study, we developed a hydrogen emitter that utilizes ultrasound‐promoted hydrogen gene therapy to prevent anesthesia/surgery‐induced cognitive impairment. Initially, FUS was employed to open the BBB, facilitating the delivery of tau siRNA‐loaded hydrogen emitters (HPPS) to the bilateral hippocampus (**Figure** **1**). The hydrogen emitter efficiently generates sufficient hydrogen, effectively scavenging hydroxyl free radicals and alleviating neuroinflammation. Additionally, HPPS releases hydrogen while undergoing structural degradation to release siRNA, thereby effectively disrupting the pathological tau cycle. This dual‐action strategy significantly enhances the prevention of anesthesia/surgery‐induced cognitive impairment. Our research indicates that in a model of POCD induced by partial hepatectomy, preoperative intravenous injection of HPPS results in a 50% reduction in hippocampal tau protein expression, alongside significant improvements in spatial recognition, object location memory, and exploratory behavior in mice. The efficacy of HPPS was further validated in a rat model, confirming the feasibility of our approach. This study develops a novel and effective treatment system aimed at preventing anesthesia/surgery‐induced cognitive impairment. It presents innovative approaches to address the challenges encountered in this field and offers new solutions for treating other neuroinflammatory conditions.

## 2 Results

### 2.1 Synthesis and Characterizations of H_2_ Emitter (HPPS) for the Delivery of Tau siRNA

**Figure 2:**
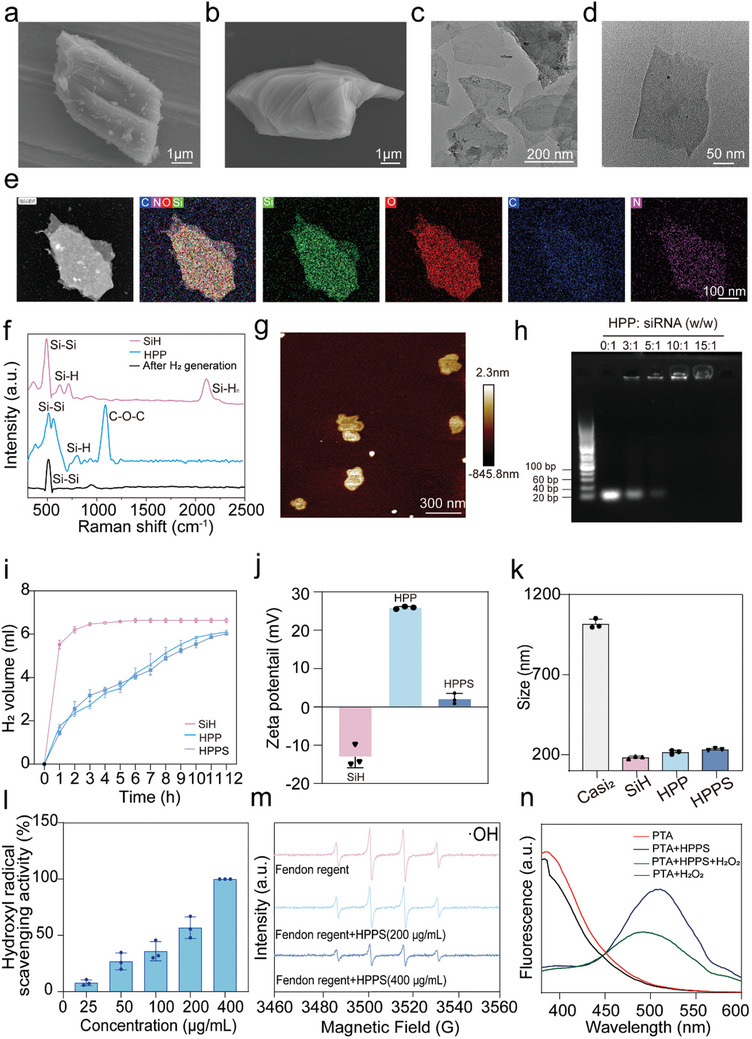
Synthesis and characterizations of H_2_ emitter (HPPS) for the Delivery of tau siRNA. a) SEM image of CaSi2 powder as the precursor. b) SEM image of SiH nanosheets with large size after HCl exfoliation. c,d) TEM image of SiH nanosheets after sonication. e) Corresponding element mappings of HPP. f) Raman spectra of SiH and HPP and the corresponding reaction product of HPP with PBS for 6 h. g) AFM image of HPP. h) Tau siRNA loading analysis at different w/w ratios of HPP to siRNA by agarose gel electrophoresis (n = 3 independent samples). i) Accumulated amount of H2 produced by SiH, HPP and HPPS in PBS, quantified by gas chromatography. j) Zeta‐ potential of SiH, HPP, and HPPS (n = 3 independent samples). k) Particle size of CaSi2, SiH, HPP, and HPPS (n = 3 independent samples). l) Hydroxyl radical‐scavenging activity of HPPS using the TMB assay (n = 3 independent samples). m) Evaluation of hydroxyl radical scavenging capability of HPPS using EPR spectroscopy (n = 3 independent samples). n) The activity of •OH consumption of HPPS evaluated by PTA (n = 3 independent samples).

In this study, we developed a H_2_ emitter that employs hydrogen‐gene therapy facilitated by ultrasound to prevent POCD. Initially, we followed established methods^[^ ^21^ ^]^ to synthesize multi‐layer SiH by etching the layered compound calcium silicide (CaSi_2_) with concentrated hydrochloric acid at ‐20 °C. According to the reaction: 3CaSi_2_ + 6HCl → 3CaCl_2_ + 6SiH,^[^ ^21^ ^]^ concentrated HCl was used to remove Ca^2+^ layers from the crystalline structure of silicide. This process removed the majority of calcium elements (Figure S1a,b, Supporting Information), forming a book‐like structure (**Figure** **2**a,b). After 36 h of advanced ultrasonic treatment and washing with anhydrous ethanol to remove impurities, few‐layer SiH was obtained, exhibiting a characteristic 2D sheet morphology (Figure 2c,d; Figure S1c, Supporting Information). To enhance the dispersion and biocompatibility of SiH in physiological environments, we further modified the nanosheets. By encapsulating SiH with mPEG‐d‐PEI at various mass ratios (Figure S2a, Supporting Information), we identified the optimal encapsulation ratio that provides the best water dispersibility, the highest stability, and allows for slow release of hydrogen. This optimal ratio was determined by assessing the preservation of the sheet structure after reacting with PBS for 6 h, as shown in the TEM images (Figure S2b, Supporting Information). The optimal mass ratio was established to be 5:1. mPEG‐d‐PEI‐coated SiH (referred to as HPP) modified at a 5:1 mass ratio exhibits excellent dispersibility in various solutions (Figure S2c,d, Supporting Information).

TEM elemental analysis of HPP verified the existence of carbon (C) and nitrogen (N) elements (Figure 2e). Raman spectroscopy was used to analyze the characteristic vibrational modes of SiH before and after modification, confirming the successful loading of the modifier with a peak at 1100 cm^−1^ assigned to the stretching vibration of the C─O─C bond (Figure 2f). The Raman spectra showed the characteristic peaks of HPP before and after 6 h of reaction with PBS, indicating the Si–H bond was no longer present in the reaction products (Figure 2f). AFM imaging showed that HPP was primarily monolayered with an average thickness of ≈1 nm (Figure 2g). In comparison to crystalline silicon, the X‐ray diffraction (XRD) patterns of SiH and HPP showed a two‐dimensional structure, which was linked to the preserved folded Si (111) planes from the CaSi_2_ crystal (Figure S3, Supporting Information).

HPP and the prepared tau siRNA were mixed for 30 min. Gel electrophoresis was employed to evaluate the siRNA binding capacity of HPP/siRNA, with a 10:1 weight ratio selected for subsequent experiments (Figure 2h).

The hydrogen bonds on the surface of SiH nanosheets facilitate efficient hydrogen release in neutral PBS. Gas chromatography was used to quantify hydrogen release, showing that HPP gradually released hydrogen over 12 h, while SiH released hydrogen completely within 1 h, confirming the sustained release performance of HPP in PBS (Figure 2i). After loading siRNA, the rate of hydrogen released from HPPS is similar to that of HPP. As expected, the average surface charge of SiH changed from −13.07 mV to 25.89 mV after mPEG‐d‐PEI modification, facilitating the attachment of siRNA with negative charges through electrostatic forces (Figure 2j). The average charge of HPP changed to 2.15 mV when the tau‐siRNA loading was successful (Figure 2j). Dynamic light scattering (DLS) measurements indicated that HPP has good dispersibility in various solutions (Figure S2d, Supporting Information), with the average particle size increasing from 183.3 nm (SiH) to 215.3 nm (HPP) in anhydrous ethanol (Figure 2k). To evaluate the stability and dispersity of SiH and HPPS in physiological conditions, the size distribution of both materials was analyzed in PBS at different time points (1, 3, 6, and 12 h). These results indicated that the PEG‐PEI surface modification of HPPS significantly enhances its colloidal stability and dispersity, making it more suitable for biomedical applications (Figure S4a,b, Supporting Information). When HPP and siRNA were successfully loaded, the average diameter of HPPS rose to 232.5 nm (Figure 2k). Additionally, under neutral conditions, siRNA bound to HPP remained undegraded after 4 h of RNase A treatment, suggesting that HPP offers protective effects for siRNA (Figure S5, Supporting Information).

Evaluating the ability of HPPS to scavenge reactive oxygen species (ROS) is essential to predict its antioxidant potential. The Fenton reaction was used to induce the production of ·OH radicals, and the TMB assay was utilized to assess the ·OH scavenging ability of HPPS, which was concentration‐dependent, with higher concentrations of HPPS exhibiting stronger ·OH scavenging activity (Figure 2l). The antioxidant capability of HPPS was demonstrated by its significant reduction in the absorbance of the ABTS radical cation (Figure S6, Supporting Information). The scavenging ability of HPPS was evaluated using electron paramagnetic resonance (EPR) spectroscopy. The results demonstrated that HPPS scavenges ·OH radicals in a concentration‐dependent manner, with higher concentrations exhibiting stronger scavenging activity (Figure 2m). Probe terephthalic acid (PTA) serves as a probe by reacting with H_2_O_2_ to generate the fluorescent compound 2‐hydroxyterephthalic acid, which exhibits a fluorescence peak at 425 nm. In the presence of HPPS, the fluorescence intensity decreased significantly, indicating that HPPS can effectively consume H_2_O_2_ (Figure 2n).

### 2.2 Protection Against ROS‐Induced Cells Damage

**Figure 3:**
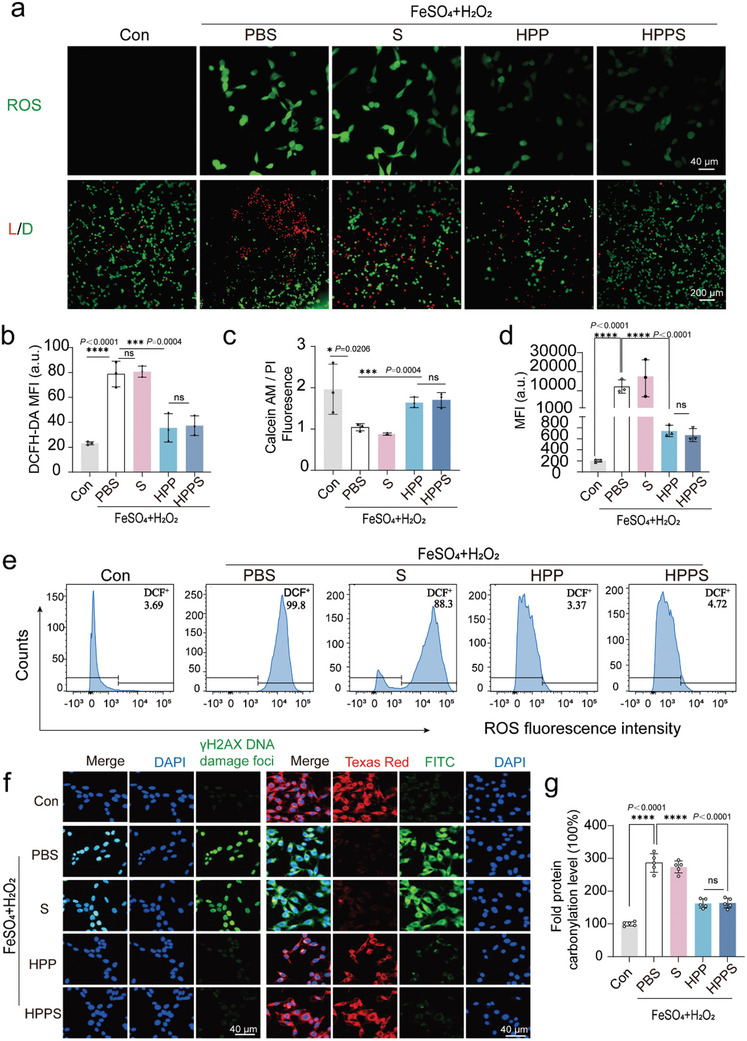
HPPS scavenges ROS and protects against ROS‐induced lipid peroxidation, DNA damage, and protein carbonylation. a) CLSM images of HT22 cells stained with DCFH‐DA and Calcein‐AM/PI (L/D represents Live/Dead) with Fenton regent and various treatments (Con, PBS, siRNA, HPP, HPPS). HPP = 200 µg mL^−1^, siRNA = 25 nm. Incubation time = 12 h. A representative image of three replicates from each group is shown (n = 3 biologically independent experiments). b) ROS fluorescence intensity of CLSM images of HT22 cells with Fenton regent and various treatments (Con, PBS, siRNA, HPP, HPPS) (n = 3 biologically independent experiments). c) Corresponding fluorescence intensity of calcein‐AM/propidium iodide (PI) of HT22 cells with Fenton regent and various treatments (Con, PBS, siRNA, HPP, HPPS) (n = 3 biologically independent experiments). d) Flow cytometry (FCM) analysis of ROS levels corresponding fluorescence intensities with Fenton regent and various treatments (Con, PBS, siRNA, HPP, HPPS) (n = 3 biologically independent experiments). e) Typical flow cytometric of ROS in HT22 cells with Fenton regent and various treatments (Con, PBS, siRNA, HPP, HPPS) (n = 3 biologically independent experiments). f) CLSM immunofluorescence images depicting γH2AX DNA damage sites in HT22 cells, as well as lipid peroxidation visualized with C11‐BODIPY581/591 staining, after treatment with Fenton reagent and various conditions (Control, PBS, siRNA, HPP, and HPPS) (n = 3 biologically independent experiments). g) Protein carbonylation level of HT22 cells after Fenton regent and various treatments (Con, PBS, siRNA, HPP, HPPS) (n = 5 biologically independent experiments). Data are presented as Mean ± SD. ****p < 0.0001, ***p < 0.001, **p < 0.01, *p < 0.05, ns: no significance.

The CCK‐8 assay was used to evaluate the safety of HPPS in HT22 and BV2 cell lines, revealing no notable cytotoxicity even at concentrations as high as 300 µg/mL (Figure S7a, Supporting Information). Exposure to Fenton reagent, which induces a high level of ROS, resulted in cell viability dropping to below 50% in both cell lines (Figure S7b, Supporting Information). However, as the concentration of HPPS increased, cell viability improved, indicating a strong protective effect against ROS‐induced damage (Figure.S7b). Confocal laser scanning microscopy (CLSM) confirmed that, following 6 h of co‐incubation with HT22 and BV2 cells (HPP = 200 µg mL^−1^; 25 nm siRNA), the majority of Cy3‐labeled HPPS had entered the cells (Figure S8a,b, Supporting Information). We further evaluated the ability of HPPS to scavenge intracellular ROS and protect cells. HPPS was found to be an effective inhibitor of ROS induced by Fenton reagent, reducing ·OH damage and cytotoxicity in HT22 and BV2 cells. When HPPS and Fenton reagent were simultaneously introduced to the culture medium, ROS production was significantly reduced, as shown by DCFH‐DA staining (**Figure** **3**a,b; Figure S9a,c, Supporting Information). Calcein acetoxymethyl ester (calcein‐AM) combined with propidium iodide (PI) staining showed the introduce of HPPS indicated a decrease in Fenton reagent‐induced apoptosis (Figure 3a; Figure S9a,b, Supporting Information). To quantify the ROS scavenging capacity, flow cytometry showed that HPPS significantly decreased the intracellular DCFH‐DA fluorescence intensity in comparison to the control group, indicating a marked decrease in reactive oxygen species levels. (Figure 3d,e; Figure S9d,e, Supporting Information). HPPS also reduced DNA damage by neutralizing ROS. We examined the ability of HPPS to protect DNA from Fenton reagent‐induced double‐strand breaks. Immunofluorescence of γ‐H2AX, a marker of DNA damage, showed a significant reduction in γ‐H2AX foci in cells pretreated with HPPS, indicating that HPPS protects against DNA double‐strand breaks (Figure 3f). Excessive ROS leads to damage of cellular biomolecules such as lipids, DNA, and proteins. The ratiometric probe C11‐BODIPY581/591, a lipophilic fluorescent dye, was utilized to detect lipid oxidation, indicated by a color change from red to green due to lipid peroxidation. As shown in Figure 3f, HPPS effectively mitigated lipid oxidative stress, inhibiting the formation of lipid peroxides. Protein carbonylation, a result of protein oxidation, reflects the extent of oxidative stress‐induced damage to proteins, which can lead to conformational changes and loss of protein function. Using the DNPH assay, we found that HPPS protected proteins from ROS‐induced accumulation of carbonyl groups (Figure 3g). The data above demonstrate the excellent ROS scavenging capability of HPP and HPPS nanosheets.

### 2.3 Intracellular Anti‐Inflammatory Effect and Gene Silencing Efficiency of HPPS

**Figure 4:**
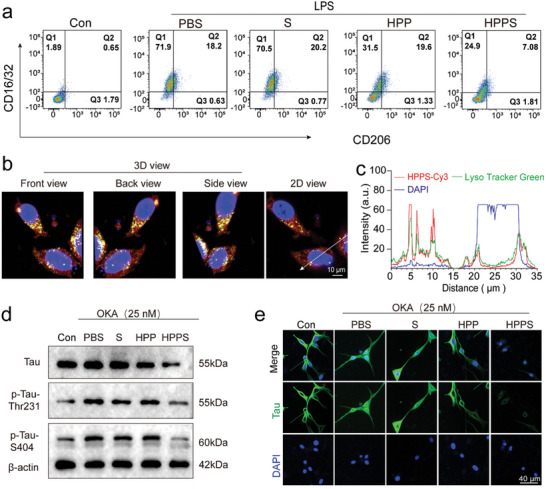
Intracellular anti‐inflammatory effect and gene silencing efficiency of HPPS. a) Typical flow cytometric analysis of BV2 cells polarization under LPS and different treatments (Con, PBS, siRNA, HPP, HPPS) (n = 3 biologically independent experiments). b) Z‐stack CLSM images of HT22 cells cultured with Cy3‐labeled HPPS nanosystem for 6 h. DAPI (blue) was used to stain the cell nuclei, LysoTracker Green (green) for endo/lysosomes, and Cy3 (red) to label HPPS. c) Corresponding fluorescence intensities in cells. d) Western blot analysis of tau and tau phosphorylation under OKA stimulation in the presence of PBS, siRNA, HPP, HPPS (n = 3 biologically independent experiments). e) Immunofluorescence analysis of tau proteins expression under OKA. HT22 cells were co‐incubated with PBS, S, HPP and HPPS.

Hydrogen demonstrates multiple biological functions, including antioxidant, anti‐apoptotic, and anti‐inflammatory activities.^[^ ^26^, ^27^ ^]^ HPPS may alleviate oxidative stress and inhibit the activation of inflammatory pathways, thereby contributing to the maintenance of microglia in a non‐polarized or M2‐polarized state. Given that microglial activation is pivotal in the pathogenesis of neuroinflammation, we aimed to elucidate whether HPP modulates the polarization state of microglia, specifically its impact on M1/M2 polarization. To this end, BV2 cells were treated with 1 µg mL^−1^ LPS to induce polarization towards M1 phenotypes. Flow cytometry was utilized to quantify the expression of the M1 marker CD16/32 and the M2 marker CD206. The phenotypic analysis of BV‐2 cells revealed that LPS stimulation resulted in an elevated M1/M2 ratio, which was significantly reduced following HPPS treatment (**Figure** **4**a, Figure S10, Supporting Information; *p* < 0.01, LPS+PBS versus LPS+HPPS). These findings indicate that HPPS effectively attenuates LPS‐induced M1 polarization of microglia. Furthermore, ELISA assays measuring TNF‐α and IL‐1β levels demonstrated that HPP mitigated LPS‐induced pro‐inflammatory responses (Figure S11, Supporting Information).

CLSM was used to examine the intracellular localization of HPPS, revealing partial colocalization of Cy3‐labeled siRNA (red fluorescence) with lysosomes (green fluorescence) and some degree of lysosomal escape, indicating successful siRNA release (Figure 4b,c; Figure S12, Supporting Information). Tau protein pathology is closely linked to chronic neuroinflammation, neurodegeneration, and cognitive impairment. Pre‐treatment with HPPS (HPP = 200 µg mL^−1^; 25 nm siRNA) for 36 h resulted in a significant knockdown of tau mRNA, achieving a 70% reduction relative to the control (Figure S13, Supporting Information). Western blot analysis and immunofluorescence further confirmed the reduced expression of tau protein (Figure 4d,e; Figure S14a, Supporting Information). HT22 cells treated with HPPS exhibited noticeably weaker green fluorescence, indicating reduced tau expression (Figure 4e). Compared to the model group (PBS + Okadaic Acid), free siRNA treatment had minimal impact on tau knockdown, whereas HPPS achieved a substantial reduction of ≈60% in tau expression (Figure S14a, Supporting Information). Okadaic Acid (OKA), a commonly used PP2A inhibitor, induces tau protein phosphorylation. After 8 h exposure of HT22 cells to 25 nm OKA, the expression levels of p‐tau‐Thr231 and p‐tau‐S404 increased to 2.4‐fold and 2.1‐fold, respectively, compared to controls (Figure 4d, Figure S14 b,c, Supporting Information). The decrease in tau levels corresponded with a marked reduction in p‐tau‐Thr231 and p‐tau‐S404 levels (Figure S14b,c). Importantly, although HPPS treatment achieved nearly 60% tau knockdown, it did not interfere with calcium influx in HT22 cells, as evidenced by comparable Fluo‐4 fluorescence intensity between the HPPS‐treated group and the PBS group (Figure S15, Supporting Information). This finding highlighted the safety profile of HPPS, indicating that its tau silencing capability does not adversely affect calcium signaling processes in neurons.

### 2.4 Evaluation and Penetration of HPPS Across BBB in Vitro and in Vivo

**Figure 5:**
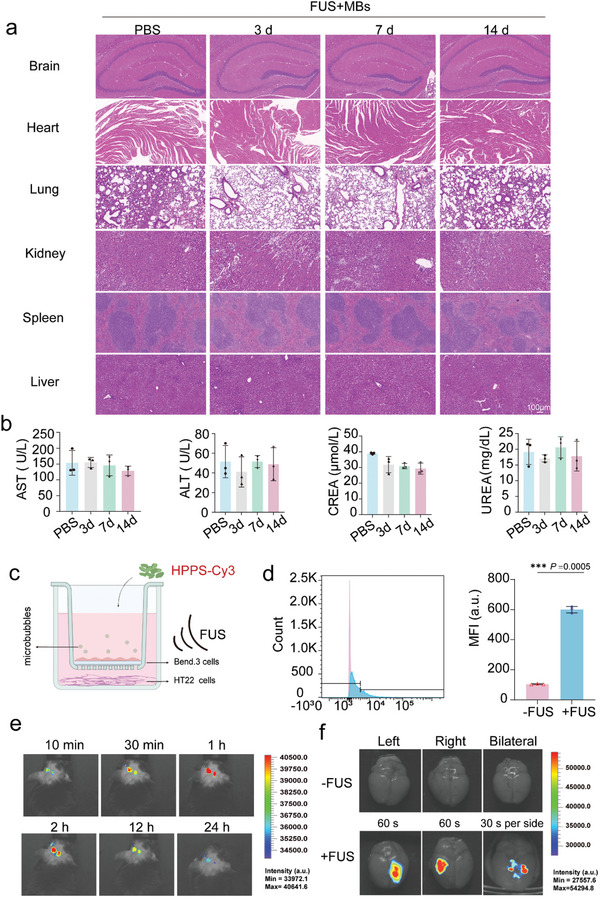
Safety and BBB penetration evaluation of HPPS in vitro and in vivo. a) HE staining of histological sections of various organs in healthy mice after receiving HPPS injection assisted with FUS+MBs within two weeks (3, 7, 14 d, HPPS = 20 mg kg^−1^, 0.1 mL) (n = 3 biologically independent samples). b) Hematological assays of mice at 3, 7, 14 d after HPPS injection (n = 3 biologically independent samples). c) Schematic representation of FUS combined with MBs to facilitate HPPS penetration of the BBB. d) Quantification of Cy3 fluorescence intensity in HT22 cells after phagocytosis of HPPS in the lower chamber of a transwell, using Flow Cytometry (n = 3 biologically independent samples). e) IVIS images of the mouse brains treated with IR783‐HPPS under the assistance of FUS and MBs within 24 h (10 min, 30 min, 1 h, 2 h, 12 h, 24 h) (n = 3 biologically independent samples). f) Ex vivo fluorescence images of the brain 1 h after intravenous injection of HPPS through the tail vein. The mice were classified into two groups: one receiving ultrasound assistance and the other without. The ultrasound‐assisted groups were further subdivided into the following conditions: ultrasound applied to the left side for 60 s, ultrasound applied to the right side for 60 s, and ultrasound applied to both sides for 30 s each (n = 3 biologically independent samples). Data are presented as Mean ± SD. ****p < 0.0001, ***p < 0.001, **p < 0.01, *p < 0.05, ns: no significance.

In this study, we evaluated the safety of HPPS. Healthy mice were administered PBS (100 µL) or HPPS (5 mg/mL, 100 µL) via tail vein injection, followed by 1 min of FUS (0.6 MPa) combined with microbubbles at a concentration of 1.5 × 10^8^/mL (100 µL) targeted to the bilateral hippocampus. Hematoxylin and eosin (HE) staining was applied to major organs, including the brain, lungs, heart, liver, spleen, and kidneys, on days 3, 7, and 14 post‐injections. The results showed no significant pathological changes in any of the major organs (**Figure** **5**a). Additionally, to evaluate the impact of HPPS on liver and kidney function, we measured several blood biochemical markers, including alanine aminotransferase (ALT), aspartate aminotransferase (AST), creatinine, and blood urea nitrogen (BUN). No significant differences were observed between the treatment and PBS control groups in these parameters (Figure 5b). These findings suggest that HPPS exhibits good biocompatibility and safety in healthy mice. Histological examination of the hippocampus, performed with HE staining, revealed no signs of microvascular rupture, indicating that FUS (0.6 MPa) combined with microbubbles is a safe procedure.

A BBB model was created with a transwell system, with mouse brain endothelioma cells (bEnd.3) cultured in the upper chamber and HT22 cells placed in the lower wells (Figure 5c). After 7 d of BBB model establishment, the transendothelial electrical resistance (TEER) reached 200 Ω cm^−^^2^ (Figure S16a, Supporting Information). The permeability coefficient for Na‐Flu in the BBB group was 7.09 ± 1.018 × 10⁻⁵ cm min^−1^, compared to 15.14 ± 1.992 × 10⁻⁵ cm min^−1^ in the control group (Figure S16b, Supporting Information). Cy3‐HPPS was added to the upper chamber, and FUS was applied through degassed water, targeting the transwell membrane (Figure 5c). The BBB model was irradiated with 0.6 MPa energy combined with microbubbles (MBs) at a concentration of 1.5 × 10⁵ mL^−1^ for 1 min. After 6 h of incubation, flow cytometry was employed to evaluate HPPS uptake in HT22 cells. As shown in Figure 5d, the average fluorescence intensity of the FUS+ group was ≈1.6 times that of the FUS‐ group, indicating that FUS+MBs enhanced BBB permeability.

In vivo IRB‐783‐HPPS (5 mg mL^−1^, 100 µL) was co‐administered with MBs (1 × 10^8^ bubbles/mL in 100 µL PBS) via intravenous injection, followed by FUS targeted to the hippocampus. FUS facilitated HPPS delivery to the hippocampus and surrounding brain regions. In Vivo Imaging System (IVIS) showed that brain fluorescence peaked 1 h after treatment (Figure 5e). Ex vivo IVIS images of the brain revealed fluorescence distribution 1 h post‐FUS, indicating that bilateral FUS irradiation (30 s unilateral irradiation,60 s in total for both sides) increased nanoparticle fluorescence intensity in the hippocampal region (Figure 5f). The reduction in ultrasound irradiation time (from 60 s to 30 s) aimed to concentrate the material's fluorescence in the hippocampus, minimizing its entry into surrounding brain regions. To further evaluate the organ distribution of HPPS following FUS‐assisted delivery, fluorescence intensities in the brain and liver were quantified. As shown in Figure S17 (Supporting Information), the mean fluorescence intensity (MFI) in the brain was significantly higher compared to the liver in both the left and right hippocampus. Specifically, the MFI in the brain was ≈1.5 times greater than that in the liver, demonstrating preferential accumulation of HPPS in the brain after FUS‐mediated delivery (Figure S17, Supporting Information).

As shown in Figure S18a,b (Supporting Information), the temperature increase in FUS‐treated mice under the parameters (0.6 MPa, 10 Hz, 10% duty cycle, 30 s) was minimal compared to controls, with both groups' temperatures stabilizing within 30 s. Similarly, in vitro experiments with HT22 cells showed less than 1 °C temperature increase, indicating negligible thermal effects (Figure S18c,d, Supporting Information). These results suggested that thermal effects are not the main mechanism for BBB opening by FUS+MB.

### 2.5 Metabolism of Hydrogen Emitters in the Brain and Tau Protein Silencing Efficiency

**Figure 6:**
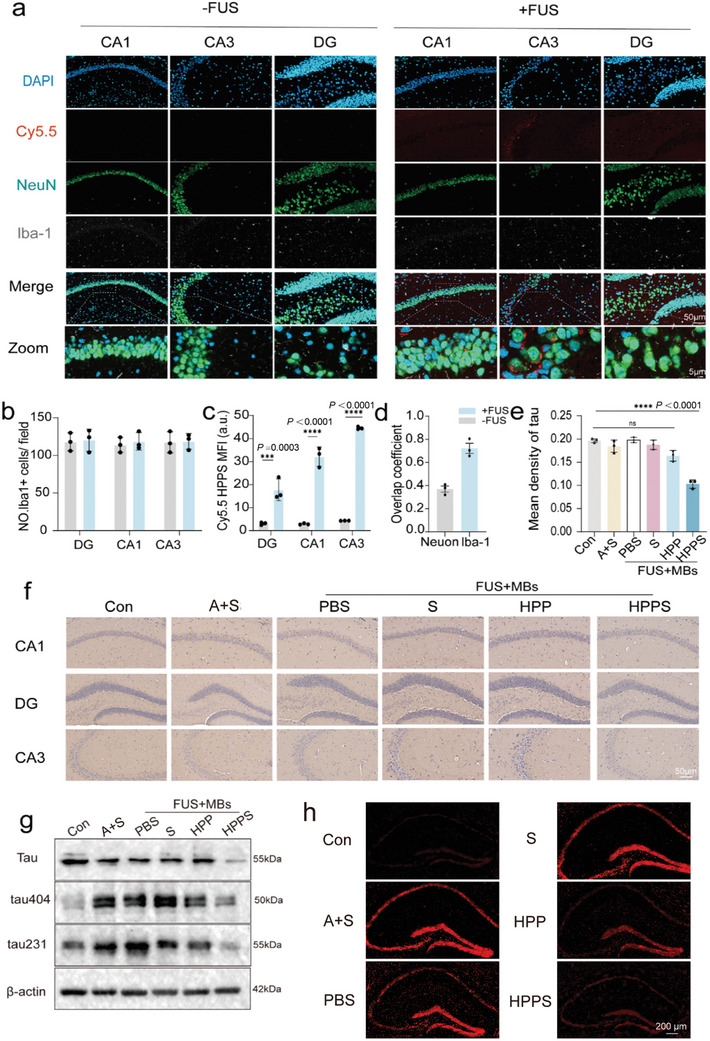
Validation of the delivery of HPPS to the hippocampus at the tissue level and its efficiency in silencing tau protein. a) Representative immunofluorescence images from mice hippocampus treated with Cy5.5‐HPPS 6 h postinjection. Neurons were identified through immunostaining for NeuN (green), while microglia were labeled with lba‐1 (gray). HPPS was labeled with Cy5.5 (red), the cell nuclei were stained with DAPI (blue) (n = 3 biologically independent experiments). b) Proportion of Iba1+ microglia in the cell population marked by DAPI. c) Mean fluorescence intensity of Cy5.5‐HPPS in different regions of the hippocampus (CA1 = Cornu Ammonis 1, CA3 = Cornu Ammonis 3, DG = Dentate Gyrus). d) Colocalization coefficient of Cy5.5‐HPPS with neurons and microglia. e,f) Immunohistochemical fluorescence quantification of tau protein in the hippocampal region and representative images at 48 h post‐injection for each group (Con, A+S, PBS, S, HPP, HPPS) (n = 3 biologically independent experiments). g) Expression of tau protein and phosphorylated tau protein in the hippocampal region of mice on the second day post‐surgery (n = 3 biologically independent experiments). h) Representative Dihydroethidium (DHE) staining of ROS fluorescence in the hippocampal CA1 region on the second day post‐surgery (n = 3 biologically independent experiments). Data are presented as Mean ± SD. ****p < 0.0001, ***p < 0.001, **p < 0.01, *p < 0.05, ns: no significance.

The metabolism of inorganic nanomaterials in the brain is characterized by slow degradation, high persistence, and significant biological stability. However, these characteristics also present certain drawbacks, such as difficulty in complete clearance, potential long‐term toxicity accumulation, and risk of neurotoxicity.^[^ ^28^ ^]^ These factors limit the safety and feasibility of using inorganic nanomaterials in neurological applications. Therefore, evaluating the clearance rate of nanomedicines from the brain is essential. Brain imaging showed that 30 s of unilateral irradiation (60 s in total for both sides) resulted in the enrichment of fluorescence intensity in the hippocampal regions bilaterally (Figure 5f). Immunofluorescence analysis of hippocampal tissue demonstrated that FUS promoted HPPS accumulation in the hippocampus without significantly increasing microglial cell numbers (**Figure** **6**a–c). Co‐localization of HPPS was higher in microglia compared to neurons (Figure 6d). We also monitored the clearance of HPPS from the brain. Immunofluorescence analysis showed that by the third day post‐administration, nanoparticle fluorescence intensity had decreased to half of that on the first day, and by the seventh day, it had dropped to less than 30% of the initial intensity. This indicated that a single administration of HPPS did not result in significant metabolic accumulation within the hippocampal or adjacent cortical regions (Figure S19, Supporting Information).

The preliminary demonstration of the therapeutic efficacy of hydrogen gene therapy was the downregulation of tau protein in the hippocampus on the second day post‐surgery, indicating the effectiveness of siRNA. Utilizing FUS, HPPS reduced hippocampal tau mRNA expression by approximately 50% at 48 h post‐injection (Figure S20a, Supporting Information). Immunohistochemical staining further confirmed that HPPS silenced tau protein expression effectively (Figure 6e,f). Western blotting showed a similar 50% reduction in tau protein levels (Figure 6g, Figure S20b, Supporting Information). The phosphorylation levels of tau protein at S404 and AT180 in the hippocampus were also decreased (Figure 6g, Figure S20c,d, Supporting Information). On the seventh day post‐surgery, the therapeutic effect of tau protein knockdown was no longer observed, as shown by Western blot analysis (Figure S21, Supporting Information).

Liver surgery may result in ROS buildup within hippocampal tissue, activating microglia, and causing secondary damage. We assessed the generation of ROS in the hippocampal region on the second day after surgery. Compared to the control group, the anesthesia and surgery group caused a significant increase in ROS levels in the hippocampal region, and this increase could be reversed by HPP and HPPS treatments (Figure 6h, Figure S22, Supporting Information).

HPPS‐mediated tau siRNA delivery, facilitated by FUS and MBs, demonstrated excellent tau protein silencing efficacy, supporting hydrogen gene therapy in reducing postoperative hippocampal neuroinflammation and cognitive impairments.

### 2.6 The Application of Hydrogen‐Gene Therapy Improved Postoperative Behavior in Neurobehavioral Tests in Mice

**Figure 7:**
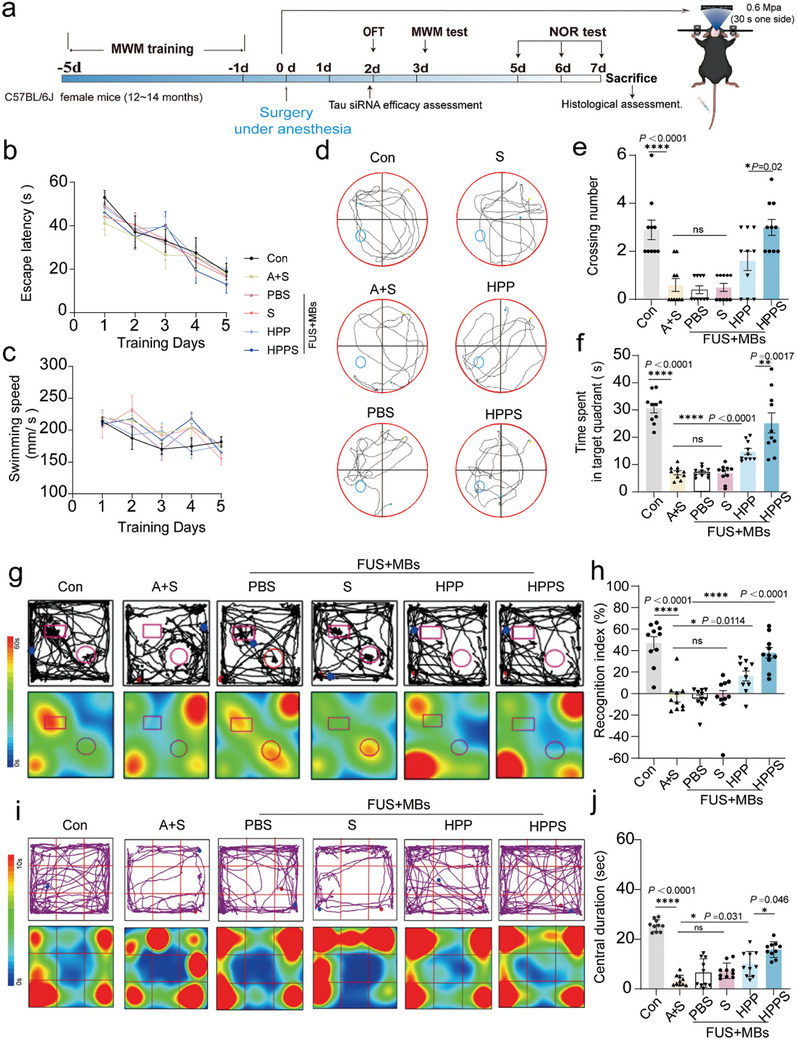
Isoflurane anesthesia and partial hepatectomy induced POCD in mice and the behavioral effects of different therapeutic drugs (PBS, S, HPP, HPPS). a) Timeline diagram of behavioral testing in mice with cognitive dysfunction following partial hepatectomy. b,c) The mean escape latency and swimming speed in the hidden platform test in the 5 d of Morris water maze (MWM) training (n = 10 biologically independent experiments). d) Representative swimming tracks of various groups. e,f) The crossing number through the platform and the staying time in the target quadrant in MWM test (n = 10 biologically independent mice) g) Representative movement tracks and time heatmap visualizations in the testing phase of NOR. h) The duration of interaction with the novel object and discrimination index (DI) in the NOR, respectively (n = 10 biologically independent mice). i) Representative movement tracks and time heatmap visualizations of open field test. j) Time spent in the central region in the open field test (n = 10 biologically independent mice). Data are presented as Mean ± SD. ****p < 0.0001, ***p < 0.001, **p < 0.01, *p < 0.05, ns: no significance.

The animal experiment protocol is outlined in **Figure** **7**a. The Morris Water Maze (MWM) test, used to assess spatial memory, demonstrated that HPPS notably enhanced both spatial learning and memory. During the water maze training conducted 5 d prior to surgery, escape latency time and speed across all groups reached similar levels with no statistically significant differences (Figure 7b,c). In the spatial probe test, where the platform was removed to assess exploration of the platform area, representative swim paths are shown in Figure 7d. The decreased duration in the target quadrant and reduced platform crossings observed in the anesthesia‐surgery group were restored by both HPP and HPPS treatments (Figure 7e,f). Thus, the MWM test indicates that partial hepatectomy impairs spatial memory in mice, while HPP and HPPS provide preventative therapeutic effects against POCD‐related spatial memory deficits.

Recognition memory, particularly dependent on the CA3 region of the hippocampus, was assessed using the Novel Object Recognition (NOR) test. The Recognition Index (RI), which reflects the proportion of time spent exploring a novel object compared to the total exploration time, was utilized to assess recognition memory. Differences in exploratory behavior were reflected in the representative tracks and time heatmaps for each group (Figure 7g), where the anesthesia‐surgery group exhibited no inclination toward the novel object location. Compared to the control group, mice in the anesthesia‐surgery group exhibited a significantly lower preference for the novel object location (Figure 7h, Figure S23, Supporting Information). These results indicate that anesthesia and surgery impair object location memory in mice. Preventive treatment with HPP and HPPS restored the Recognition Index in mice for novel object recognition. In the Open Field Test (OFT) evaluating exploration and locomotor activity, there was no significant decrease in the total distance traveled by mice in any group, indicating no significant differences in motor abilities among the groups (Figure S24, Supporting Information). However, mice in the anesthesia‐surgery group spent less time in the center area and had fewer center crossings, suggesting that mice experiencing isoflurane anesthesia and partial hepatectomy exhibit cognitive impairments and increased anxiety. Treatment with HPP and HPPS increased both the time spent in the center and the number of center crossings (Figure 7i,j). Behavioral assessments using the MWM, NOR, and OFT demonstrated that both HPP and HPPS had significant preventative effects on anesthesia/surgery‐induced cognitive impairment in mice, with HPPS showing superior efficacy.

### 2.7 FUS‐Assisted Hydrogen‐Gene Therapy Attenuated Neuroinflammation in the Hippocampus

**Figure 8:**
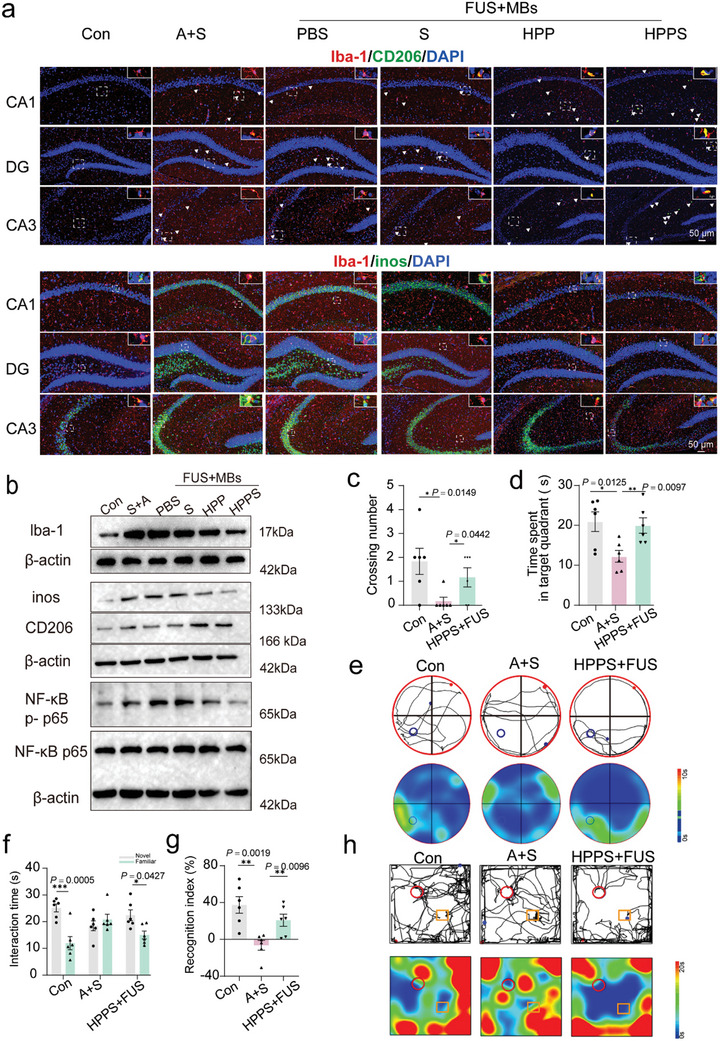
FUS‐assisted hydrogen‐gene therapy attenuated neuroinflammation in the hippocampus. a) Immunofluorescence of CD206 (M2 marker, green) and inos (M1 marker, green) in hippocampus. Iba1 (microglia marker) were stained red and nuclei were stained blue (Con, A+S, PBS, S, HPP, HPPS) (n = 3 biologically independent experiments). b) Western blot of lba‐1, inos, CD206, NF‐κB p65, NF‐κB p‐p65 of different groups (Con, A+S, PBS, S, HPP, HPPS) (n = 3 biologically independent experiments). c,d) The number of platform crossings and the duration spent in the third quadrant during the MWM test for the different experimental groups (Con, A+S, HPPS+FUS) (n = 6 biologically independent rat). e) Representative swimming tracks and time heatmap visualizations in the MWM test of various groups (Con, A+S, HPPS+FUS). f,g) The duration of interaction with both the novel and familiar objects during the NOR test across different groups (Con, A+S, HPPS+FUS) (n = 6 biologically independent rat). h) Representative movement tracks and time heatmap visualizations in the testing phase of NOR (Con, A+S, HPPS+FUS). Data are presented as Mean ± SD. ****p < 0.0001, ***p < 0.001, **p < 0.01, *p < 0.05, ns: no significance.

As shown in **Figure** **8**a, this study examined the expression of iNOS and CD206, markers for M1 and M2 microglia respectively, in hippocampal tissue from mice 7 d after surgery using immunofluorescence double staining. Compared to the control group, the surgery group exhibited significantly increased Iba‐1 fluorescence intensity indicative of microglial activation in the hippocampus, confirmed by the Western blot analysis of Iba‐1(Figure 8b). Immunofluorescence double staining further distinguished the activated microglial cell phenotypes, revealing that compared to the control group, the anesthesia and surgery (A+S) group had a significant increase in Iba1^+^/CD206^+^ cells in the CA1, CA3, and DG regions of the hippocampus (Figure 8a, Figure S25a, Supporting Information), as well as an increase in Iba1^+^/iNOS^+^ cells (Figure 8a, Figure S25b, Supporting Information). Pre‐treatment with HPP or HPPS significantly reduced the number of Iba1^+^ cells in the hippocampus (Figure 8a, Figure S25c, Supporting Information). Also, HPPS pre‐treatment significantly decreased the percentage of Iba1^+^/iNOS^+^ microglia while increasing the percentage of Iba1^+^/CD206^+^ microglia (Figure S25a,b, Supporting Information). This was further confirmed by western blot analysis (Figure 8b, Figure S26a,b, Supporting Information). High levels of ROS can induce and sustain M1 polarization in microglia.^[^ ^29^ ^]^ Previous research has shown that the interaction between tau aggregates and microglial receptors, such as toll‐like receptors, leads to NF‐κB activation, promoting the transcription of pro‐inflammatory genes and perpetuating the cycle of neuroinflammation.^[^ ^30^ ^]^ The NF‐κB signaling pathway, a key regulator of the pro‐inflammatory (M1) microglial phenotype,^[^ ^31^ ^]^ was also investigated. Western blotting showed significant upregulation of this pathway in the A+S group, downregulation in the HPP group, and further suppression in the HPPS group (Figure 8b; Figure S26b, Supporting Information). These findings suggest that HPPS mitigates microglia‐mediated neuroinflammation in the POCD model, inhibits M1 polarization, and promotes M2 polarization of microglia in the hippocampus, thereby reducing neuroinflammation.

On the second day following surgery, we confirmed that HPPS inhibits the levels of two kinds of phosphorylated tau proteins in the hippocampus (Figure 6g). HPPS demonstrated superior efficacy over HPP in inhibiting neuroinflammation, likely due to the knockdown of tau protein, which disrupts the pathological cycle of tau. M1 polarization of microglia is typically linked to the production of pro‐inflammatory cytokines that worsen neuronal damage and impair cognitive function. ELISA was used to assess the expression of these cytokines in hippocampal tissue to evaluate the impact of HPPS on inflammatory protein levels (Figure S27a–c). In comparison to the control group, the A+S group exhibited increased levels of pro‐inflammatory cytokines, specifically IL‐6, IL‐1β, and TNF‐α. Conversely, both HPP and HPPS notably decreased the expression of these inflammatory mediators, with HPPS showing greater efficacy (Figure S27a–c). We also assessed the activation of astrocytes in the hippocampal CA1 region by quantifying the number of GFAP+ cells using immunofluorescence staining (Figure S28, Supporting Information). Compared to the control group (Con), the A+S group exhibited a significant increase in the number of GFAP+ astrocytes, indicative of heightened astrocyte activation. However, treatment with HPPS markedly reduced the number of GFAP+ cells compared to the A+S group, demonstrating its ability to mitigate astrocyte activation (Figure S28, Supporting Information). Based on the above results, it can be preliminarily inferred that FUS‐assisted hydrogen emitter carrying tau siRNA can alleviate hippocampal neuroinflammation following partial hepatectomy.

### 2.8 Cognitive Effects of Hydrogen Gene Therapy on Rats After Partial Hepatectomy

To further validate the preventive and therapeutic effects of HPPS on anesthesia/surgery‐induced cognitive impairment, we conducted an efficacy re‐evaluation in 10‐month‐old Sprague‐Dawley rats undergoing partial hepatectomy. The study workflow is depicted in Figure S29 (Supporting Information). By intravenously injecting rats with HPPS (HPPS = 20 mg kg^−1^) and using FUS to open both hippocampi, we validated the knockdown efficiency of three kinds of tau‐siRNA sequences via immunofluorescence. At 48 h post‐administration, siTAU2 showed the best effect (Figure S30, Supporting Information). Western blot analysis demonstrated a significant reduction in tau protein levels in the siTAU2 group compared to the control group, while siTAU3 showed moderate effects (Figure S30, Supporting Information). Therefore, siTAU2 was selected for subsequent experiments. Five days prior to surgery, MWM training was conducted, with no significant statistical differences observed in swimming speed and escape latency among the Con, A+S, and HPPS+FUS groups (Figure S31a,b). On the third day post‐surgery, spatial exploration tests were initiated. The MWM test revealed a decrease in both the frequency of platform crossings and the duration spent in the third quadrant in the model group, while HPPS pre‐treatment restored spatial recognition ability (Figure 8c–e). In the NOR test, the model group exhibited less interaction time with the novel object in comparison to the familiar one (Figure 8f–h). Ultrasound‐assisted hydrogen emitters markedly enhanced object location recognition in rats when compared to the model group (Figure 8f–h), indicating that surgery and anesthesia impaired object recognition, while hydrogen gene therapy mitigated anesthesia/surgery‐induced cognitive impairment.

## 3 Discussion

POCD affects thousands of patients. It impairs memory, attention, and executive function. However, there is a lack of effective treatments. The pathogenesis of POCD is driven by a complex interaction among phosphorylated tau, ROS, and neuroinflammatory processes. ROS plays a crucial role in regulating microglial polarization. Elevated ROS levels promote a pro‐inflammatory microglial state, worsening neuroinflammation.^[^ ^32^ ^]^ Pro‐inflammatory microglia enhance tau phosphorylation in nearby neurons.^[^ ^33^ ^]^ Hyperphosphorylated tau detaches from microtubules and aggregates into oligomers, forming neurofibrillary tangles. Accumulated tau aggregates are secreted into the extracellular space, triggering tau pathology.^[^ ^13^ ^]^ Studies show that reactive microglia are linked to the spread of pathological tau in the brain.^[^ ^13^, ^34^ ^]^ The cycle of inflammation and tau phosphorylation creates a vicious feedback loop that amplifies neurodegeneration.^[^ ^35^ ^]^ Breaking this cycle requires inhibiting tau phosphorylation and eliminating excess ROS to reduce neuroinflammation. To disrupt the vicious cycle between tau protein and ROS, we developed an ultrasound‐assisted hydrogen emitter to enable hydrogen‐based gene therapy for the prevention of anesthesia/surgery‐induced cognitive impairment.

H_2_ has been shown to be beneficial in addressing various health issues, including malignancies, heart diseases, neurodegenerative disorders, metabolic disorders, diabetes, and dermatological lesions.^[^ ^17^ ^]^ However, the high diffusivity and limited solubility of H_2_ present challenges in concentrating at specific targets, limiting its therapeutic efficacy. The integration of hydrogen with nanomaterials offers a potential solution to this limitation.^[^ ^36^ ^]^ SiH, a hydrogenated two‐dimensional nanosheet, effectively reduces inflammation in local tissues^[^ ^21^ ^]^ and colitis.^[^ ^37^ ^]^ This suggests that hydrogen therapy via SiH is effective in suppressing inflammation. Nonetheless, the use of SiH nanosheets in biomedicine is constrained by their poor solubility and dispersibility in physiological environments. To enhance dispersibility and biocompatibility, we modified SiH with mPEG‐d‐PEI in different ratios. This approach achieved well‐dispersed nanosheets capable of gradually releasing hydrogen over 6 h, meeting the requirements for treating acute inflammation.

Previous studies show that reducing tau phosphorylation or clearing tau aggregates can attenuate microglial activation and reduce neuroinflammation.^[^ ^38^ ^]^ Reducing total tau protein has been shown to decrease its aggregation, showing therapeutic effects.^[^ ^39^ ^]^ In this study, siRNA therapy was employed to transiently reduce the total tau levels during the perioperative period. siRNA therapy offers the advantage of highly specific gene silencing, allowing targeted modulation of disease‐related genes. Despite the potential of siRNA drugs, their development faces key challenges. These include targeted accumulation, cellular uptake, endosomal and lysosomal escape, and drug performance.^[^ ^40^ ^]^ In this study, we delivered siRNA using nanosheets. mPEG‐d‐PEI serves a dual role: it modifies SiH to enhance its biocompatibility and dispersibility, allowing for controlled hydrogen release, and it alters the charge of SiH to positive, facilitating the binding of negatively charged siRNA. HPP demonstrates excellent siRNA protection. Additionally, PEI, a cationic polymer linker, enhances solubility, improves cellular uptake, and facilitates lysosomal escape, thus increasing therapeutic efficacy.^[^ ^41^ ^]^ HPPS demonstrates effective tau silencing in both cellular and tissue environments.

In fact, the delivery of inorganic nanomedicines into the brain remains a major challenge, primarily due to the restrictive nature of the BBB which impedes the transport of nanodrugs. In our study, we overcame the BBB using ultrasound assistance, which enhanced drug accumulation in the bilateral hippocampus. Microbubble‐assisted FUS can transiently open the BBB, providing a minimally invasive method for targeted drug delivery to the brain.^[^ ^24^, ^42^ ^]^ In vivo imaging showed that brain fluorescence peaked 1 h after FUS irradiation. We also assessed the safety of FUS‐induced BBB opening, ensuring no microhemorrhages occurred in the mouse brain. With the assistance of FUS, we successfully delivered HPPS via tail vein injection into the hippocampus. These nanosheets were partially phagocytosed by microglia and neurons, achieving effective tau knockdown and inhibition of phosphorylated tau. This suppressed tau pathology. The NF‐κB signaling pathway was downregulated, and microglial polarization shifted from M1 to M2. This reduced neuroinflammation and improved behavior and cognition. Our research demonstrates that dual‐targeting strategy by rebalancing microglial polarization can effectively mitigate neuroinflammation. This study offers a new approach for preventing anesthesia/surgery‐induced cognitive impairment.

In conclusion, our utilization of ultrasound‐assisted hydrogen emitters for hydrogen‐gene therapy represents an efficient and innovative strategy to prevent anesthesia/surgery‐induced cognitive impairment. By disrupting the detrimental cycle of tau protein and ROS, this approach can significantly mitigate postoperative neuroinflammation in the hippocampus. The implications of this treatment extend beyond reducing postoperative complications, it also contributes to improving patients' overall quality of life. Furthermore, this treatment strategy offers a novel perspective for addressing neuroinflammatory diseases and may foster advancements in the field of brain science.

## 4 Experimental Section

### Chemicals

CaSi_2_ was purchased from Alfa Aesar. All other chemicals and test kits, unless otherwise stated, were sourced from Sigma–Aldrich. Dulbecco's modified eagle's medium (DMEM, high glucose), fetal bovine serum (FBS), PBS, and penicillin/streptomycin were all obtained from YoBiBio (Shanghai, China). The anti‐tau antibody (A0002), anti‐tau (404) antibody (AP0170), anti‐β‐actin antibody (AC038), and HRP‐conjugated against rabbit or mouse (AS065) was gained from abconol. Anti‐lba‐1‐ antibody (17 198), anti‐ p65‐antibody (8242), anti‐p‐p65 antibody (3033), and anti‐tau (231) (71 429) antibody obtained from CST, while anti‐inos (PA1‐036) and anti‐CD206 (MA5‐16872) antibodies were sourced from Thermo Fisher. Dihydroethidium (DHE) was purchased from Invitrogen‐Molecular Probes (Eugene).

### Characterizations

The morphology of CaSi_2_ was observed by SEM (ZEISS GeminiSEM 300). The morphology and element mapping of SiH nanosheets was obtained by Transmission electron microscopy (TEM) (JEM‐2100F, JEOL). Nano ZS 960 Malvern Zetasizer Nanoseries instrument (Malvern Instruments Ltd., UK) was used to measure dynamic light scattering (DLS) and zeta potential. XRD was tested with a Bruker D8 X‐ray diffractometer. Confocal laser scanning microscopy (CLSM, ZEISS LSM900) was applied to capture confocal images. A flow cytometer (BD LSR Fortessa) was used to perform FCM.

### Synthesis of SiH

0.5 g CaSi_2_ was dissolved in 100 mL concentrated aqueous HCl solution at −20 °C, using a magnetic stirrer set at 600 rpm for 10 d. The green product was separated by centrifugation at 10 000 rpm for 10 min and subsequently washed four times with ethanol. The products were dispersed in 50 mL ethanol, followed by probe ultrasound in ice bath for 36 h. The mixture was then centrifuged at 15 000 rpm for 10 min to isolate the SiH nanosheets with the desired small lateral size.

### Fabrication of mPEG‐PEI‐Coated SiH

mPEG‐CHO was synthesized following the method described in a previous study.^[^ ^43^ ^]^ mPEG‐d‐PEI was synthesized by mixing PEI and mPEG‐CHO in a 2:1 weight ratio at pH 7.4 for 30 min. Different quality of SiH was added to the resulting solution to prepare the mPEG‐d‐PEI‐coated SiH. The resulting mPEG‐PEI‐coated SiH was then mixed with tau siRNA and incubated for 10 min to produce SiH@PP/siRNA (HPPS).

### Agarose Gel Electrophoresis

The HPPS delivery nanosystem was generated after co‐incubating HPP/siRNA in various mass ratios (0:1, 3:1, 5:1, 10:1, and 15:1). After centrifugation, the sediment was loaded onto a 2% (w/v) agarose gel. Electrophoresis parameters was set at 80 V for 25 min. The samples were then visualized using a UV illuminator and the Gel Doc System (Bio‐Rad). To evaluate the resistance of HPPS to degradation in an RNase environment, siRNA (0.5 µg) alone or with HPP was co‐incubated with RNase. Sodium dodecyl sulfate (SDS) at a final concentration of 2% was used at the indicated times to promote siRNA release.

### The Stability of HPPS Structure

To achieve improved biocompatibility and controlled hydrogen release, different mass ratios of SiH were combined with PP. The resulting HPPS mixtures were combined with PBS, medium and physical photos were taken at several time points (1 min, 1 h, 3 h, and 6 h) to determine the most suitable ratio. Additionally, TEM images of the product after 6 h of reaction with PBS were captured to observe the residual structure. Once the optimal ratio was identified, its stability and dispersibility were assessed in various solutions (PBS, water, serum‐free medium, FBS) and further analyzed using DLS.

### Hydrogen Release of HPP

The quantitative analysis of gaseous H_2_ accumulated during the reaction of SiH and HPPS with PBS was conducted using gas chromatography. The hydrogen collection device is referred to the article by Yanling You et al.^[^ ^21^ ^]^

### ·OH Scavenging Activity

TMB was selected as the chromogenic substrate to detect the content of ·OH. H_2_O_2_ (10 mm) and FeSO_4_ (4 mm) were first reacted for 0.5 h, then HPPS nanosheets at different concentrations (0, 25, 50, 100, 200, 400 µg mL^−1^) were added for 0.5 h, and finally, TMB (0.6 mm) was added. The absorbance was measured at 652 nm (n = 3). The total antioxidant capacity of HPPS was measured by using a TAC assay kit based on ABTS radical cation (ABTS+) decolorization method according to the manufacture's instruction. ESR spectroscopy was employed to assess the ·OH scavenging activity of HPPS using DMPO as the trapping agent. Fenton reagent was mixed with HPPS (200 and 400 µg mL^−1^) in PBS (pH 7.4) and incubated at 37 °C for 5 min, followed by the addition of DMPO (25 mm).

H_2_O_2_ (10 mM) decomposes into ·OH, which are further captured by PTA (500 µm) to generate highly fluorescent TAOH, exhibiting an emission peak around 425 nm. The elimination of H_2_O_2_ was assessed by monitoring the fluorescence signal of TAOH. The consumption of H_2_O_2_ (10 mM) with HPPS (200 µg/mL) was determined using a UV–vis–NIR spectrophotometer (n = 3 for each group).

### Cell Culture and Mice

The mouse macrophage cell line (BV2), mouse hippocampal neuronal cell line (HT22), and mouse brain microvascular endothelial cell line (bEnd.3) were obtained from the China Center for Type Culture Collection (Wuhan, Hubei, China). These cells were cultured in DMEM supplemented with 1% penicillin‐streptomycin and 10% fetal bovine serum (FBS) at 37 °C in a 5% CO_2_ atmosphere. Female C57BL/6J mice (12–14 months old) were purchased from Ling Chang Biological Technology Co., Ltd. The mice were housed in a humidity‐ and temperature‐controlled room with a 12:12 h light/dark cycle and had free access to laboratory food and water. All animal procedures were carried out following protocols in compliance with the National Ministry of Health regulations and received approval from the Laboratory Animal Center at Shanghai Tenth People's Hospital (Approval No. SHDSYY‐2023‐82278).

### Cell Viability Assay

Using the cell counting kit‐8 (CCK‐8), BV2 and HT22 cells were incubated with the CCK‐8 solution for 2 h, and their viability was assessed at 450 nm.

### Analysis of the Cellular Uptake and Lysosomal Escape of HPPS Nanoparticles

The Cellular Uptake of Cy3‐labeled HPPS was analyzed using CLSM. The BV2 and HT22 cells were plated onto a confocal focusing dish at the density of 1 × 10^5^ cells for 8 h. The HPPS in the medium was introduced in the BV2 and HT22 cells and incubated (HPP = 200 µg mL^−1^, siRNA = 25 nm) for different durations (1, 3, 6 h). Then DAPI (10 µg mL^−1^) and Lysotracker (0.5 µg mL^−1^) was added respectively.

### ROS Scavenging and Cytoprotection

HT22 and BV2 cells were seeded into 20 mm glass‐bottom cell culture dishes at a density of 1 × 10^5^ cells per dish. To induce ROS production, Fenton's reagent (400 µm H_2_O_2_ and 40 µm FeSO_4_) was added to the culture medium. Simultaneously, PBS, HPP, siRNA, or HPPS (HPP = 200 µg mL^−1^, siRNA = 25 nm) was introduced as therapeutic agents and incubated for 12 h. Specifically, the oxidant‐sensitive fluorescent dye DCFH‐DA was used as a ROS probe for cellular ROS imaging. Additionally, the cellular ROS levels of stained cells after different treatments were analyzed by flow cytometry. Furthermore, CLSM was employed to examine the live and dead conditions of HT22 and BV2 cells after treatment, using calcein‐AM and PI to stain live and dead cells, respectively.

### Protection for the Three Components in the Cell

Assessment of protein carbonylation, lipid peroxidation, and DNA damage were measured by DNPH assay, C11‐BODIPY^581/591^ dye, phospho‐histone‐H2AX immunofluorescence staining, respectively.

### BV2 Polarization

BV2 cells were induced with LPS (1 µg mL^−1^) and treated with siRNA, HPP, and HPPS (HPP = 200 µg mL^−1^, siRNA = 25 nm) for 12 h. BV2 cells were stained in 1% BSA‐PBS buffer for 20 min at room temperature. The following antibodies were used: APC‐CD16/32 and PE‐CD206 (biolegend). Flow cytometry was employed to analyze the M1ratios. Additionally, the concentrations of IL‐1β and TNF‐α in the hippocampus and medium were measured using ELISA kits according to the manufacturer's instructions.

### Gene Silencing In Vitro

Tau siRNA (mouse): 5′‐CCU AGA AAU UCC AUG ACG AUU‐3′; tau siRNA (rat): siTAU1:5‐GCAUGUGACUCAAGCUCGA‐3; siTAU2: 5‐AGUUAGGGACGAUGCGGUA‐3; siTAU3:5‐GAUAGAGUCCAGUCGAAGA‐3; HT22 cells were treated with OKA (25 nM) for 8 h to establish a phosphorylation model. The cells were then divided into five groups: control, OKA, OKA + siRNA alone, OKA + HPP, and OKA + HPPS (at 25 nm concentration of siRNA). After 36 h of incubation, total cellular RNA was extracted using TriZol following the manufacturer's protocol, and total protein was collected after lysis with RIPA buffer. The expression of the target gene tau was detected using real‐time fluorescence quantitative PCR and immunofluorescence. Additionally, Western blot analysis was employed to detect the expression of the target protein and phosphorylated tau protein. The primers used were as follows: Tau forward primer:5′‐TTTGACACAATGGAAGACCATGC‐3′; Tau reverse primer: ‐5′ GCCACACGAGCTTTTAAGCC‐ 3′

### FUS‐BBB Opening In Vitro

The TEER of the BBB model was measured from days 1 to 7 using the fully automated CellZscope device, with the cell‐free transwell chamber serving as a blank control. TEER (Ω cm^2^) = (Resistance value of the experimental group – Resistance value of the blank group) × Membrane area (cm^2^). bEnd.3 cells were seeded on the bottom side of a Transwell culture insert with a 0.4‐µm pore polycarbonate membrane at a density of 700 000 cells per well. TEER was measured daily using an epithelial volt‐ohmmeter until it plateaued around 80 ohm·cm^2^ after 1 week, at which point permeability experiments were conducted. The amount of Na‐flu passing through the BBB model was measured using a microplate reader, and its permeability was calculated using the Artursson formula. HT22 cells were then seeded in the lower layer of the chamber, and FUS was applied using a transducer amplified by a power amplifier to penetrate the Transwell membrane. After 4 h, Cy3‐HPPS (HPP = 200 µg mL^−1^, siRNA = 25 nm) fluorescence intensity in HT22 cells was detected by flow cytometry.

### In Vivo Safety Evaluation

Mice (n = 9) were intravenously injected with HPPS in physiological saline (25 mg kg^−1^, 5 mg mL^−1^), while control mice (n = 3) received PBS, both groups received FUS + MBs (1 × 10^8^ bubbles mL^−1^ in 100 µL of PBS). At 3, 7, 14 d post‐treatment, major organs of the HPPS group were removed, fixed, and stained with HE for microscopic assessment. Blood samples were collected to evaluate liver and kidney function parameters. The PBS group was used as a control, with organs and blood samples collected on the third day after administration.

### FUS‐BBB Opening In Vivo

To investigate BBB opening using FUS, a FUS transducer (1.0 MHz, 38 mm diameter) was operated by a function generator connected to a power amplifier. The transducer, housed in a cone filled with degassed water, directed the FUS beam into the brain with parameters set at 0.6 MPa acoustic pressure, 10 Hz pulse repetition frequency, and 10% duty cycle. When unilateral irradiation was applied, the ultrasound duration was set to 60 s; for bilateral irradiation, the duration was limited to 30 s per side. The coordinates of the mouse hippocampus relative to the bregma are: AP (anterior–posterior) −2.0 mm, ML (medial‐lateral) ±1.5 mm, and DV (dorsal‐ventral) −1.5 mm. Mice, prepared by shaving and depilating their heads and securing them in a stereotaxic frame, were injected with IRB783‐labeled HPPS (25 mg kg^−1^, 5 mg mL^−1^, siRNA = 100 nmol kg^−1^) and microbubbles (1 × 10^8^ bubbles mL^−1^ in 100 µL of PBS) before being exposed to FUS. Brain imaging was performed using spectrum imaging system at 10 min, 30 min, 1 h, 2 h, 12 h, and 24 h after FUS. The imaging data were processed and analyzed using IVIS Living Image software.

The coordinates of the rat hippocampus relative to the bregma are: AP (anterior‐posterior) −3.0 mm, ML (medial‐lateral) ±2.0 mm, and DV (dorsal‐ventral) −3.0 mm. The ultrasound irradiation intensity for both sides of the rat hippocampus was set to 0.6 MPa, with the duration increased to 1 minute per side (HPPS = 20mg kg^−1^). Infrared thermal imaging was used to monitor the skin surface temperature of the treated (FUS+) and untreated (FUS−) areas immediately after ultrasound exposure. Thermal images were captured using an infrared thermal imager, and the temperatures were recorded at the site of treatment.

### Anesthesia and Partial Hepatectomy Surgery

Anesthesia was administered and maintained in a dedicated chamber with 3% isoflurane in 60% oxygen. After 30 min of exposure to isoflurane, the abdominal area of the mouse was shaved and disinfected with povidone iodine. The surgical procedure, which took about 30 min, involved making a 1 cm midline incision to access the peritoneal cavity. The median and left lobes of the liver (comprising 70% of the total liver) were tied at their base with 4‐0 sterile sutures.^[^ ^44^ ^]^ The lobes were then excised using scissors, and the wound was observed to ensure there was no significant bleeding. The peritoneal lining and skin were closed, and polysporin was applied to the wound to prevent infection. A heating pad was used to keep the body temperature between 36 and 37 °C during surgery. Buprenorphine (0.1 mg kg^−1^) was given after the induction of anesthesia and before the incision to alleviate pain. Control mice did not undergo anesthesia or surgery. The liver resection procedure in rats is similar to that in mice.

### Animal Behavior Experiment—Morris Water Maze (MWM)

Following the protocol established by Ariel K. Frame et al.,^[^ ^45^ ^]^ the Morris water maze (MWM) was used to assess spatial learning and memory. The experiment involved a smaller pool, 120 cm in diameter, and 30 cm in height, with a hidden platform placed underwater. Training trials were conducted four times daily across 5 days, followed by a 60 s test to locate the platform. For experiments with rats, a larger pool, measuring 180 cm in diameter and 50 cm deep, was employed to suit the study's needs.

### Novel Object Recognition (NOR)

The novel object recognition (NOR) test was conducted with modifications,^[^ ^46^ ^]^ involving three phases: habituation, training, and testing. During habituation (day 1), mice adapted to an empty chamber for 5 min. In the training phase (day 2), mice explored two identical objects in the chamber for 5 min. On the testing day (day 3), one familiar object was replaced with a novel object, and mice were observed for 5 min to calculate the discrimination index (DI), which measures their interaction time with the novel versus familiar objects using Visutrack software.

### Open Field Test

The OFT was examined in a box (50 cm × 50 cm × 50 cm) according to the method of Junxing Ma et al.^[^ ^47^ ^]^ The immobility time and time spent in the center zone was automated by Visutrack software ((XinRuan Information Technology Co., Shanghai, China).

### DHE Staining

Intracellular ROS levels in the hippocampal region were assessed using in situ DHE fluorescence staining. Tissue sections were treated with a 1 µm DHE solution diluted in PBS and incubated at room temperature for 40 min. Image density was analyzed using ImageJ software.

### Immunofluorescence Staining

To understand microglial activation and polarization in the mouse hippocampus, M1 and M2 markers were detected by immunofluorescence. Brain coronal slices (20 µm thick) were incubated with 5% goat serum to block non‐specific binding, followed by incubation overnight with anti‐Iba‐1 and rabbit anti‐CD206 antibodies or anti‐inos at 4 °C.

### Western Blot Analysis

Proteins were extracted from the hippocampus of mice and HT22 cells for immunoblotting analysis. The concentration of the extracted proteins was quantified using the Bio‐Rad Protein Assay with bovine serum albumin (BSA) as a standard. Equal amounts of each protein sample were loaded onto SDS‐PAGE gels for separation, then transferred to PVDF membranes. These membranes were incubated overnight at 4 °C with primary antibodies, followed by the application of fluorescent secondary antibodies.

### Statistical Analysis

Data were shown as means ± SD. Statistical analyses were calculated through GraphPad Prism software (version 9.0.0, GraphPad Software, San Diego, California, USA). For multiple group comparisons, one‐way analysis of variance (ANOVA) was used. For two groups comparisons, unpaired Student's *t*‐test was used. *p < 0.05, **p < 0.01, ***p < 0.001, and ****p < 0.0001, ns, *p* >0.05.

## Conflict of Interest

The authors declare no conflict of interest.

## Author Contributions

R. Z., Y. F., and C. L. contributed equally to this work.

## Supporting information



Supporting Information

## Data Availability

The data that support the findings of this study are available on request from the corresponding author. The data are not publicly available due to privacy or ethical restrictions.
