# Supplementary material for: Ultrasound‐Assisted H2 Transmitter Enables Hydrogen‐Gene Therapy to Prevent Anesthesia/Surgery‐Induced Cognitive Impairment
Source: Adv Sci (Weinh). 2025 Mar 6;12(17):2414397. doi: 10.1002/advs.202414397 (PMC12061270; doi:10.1002/advs.202414397)
Supplement: Supplementary file 1 — Supporting Information [file ADVS-12-2414397-s001.docx]

Supporting Information

**Ultrasound-assisted H_2_ transmitter enables hydrogen-gene therapy to prevent anesthesia/surgery-induced cognitive impairment**

Ruonan Zhan ^1#^, Yan Fang^4#^, Chuyun Lou^5#^, Nan Chen^1^, Xuan Mo^1^, Bo Jiao^1^, Mengke Liu^1^, Yangxi Zhao^1^, Weichen Xu^6^, Huixiong Xu ^2*^, Haohao Yin^2,3*^&Yi Zhang^1*^

^1^Department of Anesthesiology and Pain Medicine, Hubei Key Laboratory of Geriatric Anesthesia and Perioperative Brain Health, and Wuhan Clinical Research Center for Geriatric Anesthesia, Tongji Hospital, Tongji Medical College, Huazhong University of Science and Technology.

^2^Department of Ultrasound, Institute of Ultrasound in Medicine and Engineering, Zhongshan Hospital, Fudan University, Shanghai, 200032, P. R. China.

^3^Department of Ultrasound, Zhongshan Hospital (Xiamen), Fudan University, 361015, P. R. China.

^4^Department of Ultrasound, Huashan Hospital, Fudan University, Shanghai 200040, China.

^5^Department of Radiology, The First Affiliated Hospital of Zhengzhou University, No.1, Eastern Jianshe Road, Zhengzhou, 450052, Henan Province, China.

^6^Department of Medical Ultrasound, Center of Minimally Invasive Treatment for Tumor, Shanghai Tenth People’s Hospital, School of Medicine, Tongji University, Shanghai 200072, China.

^#^These authors contributed equally

*Corresponding author email: yi_zhang18@tjh.tjmu.edu.cn;

yin.haohao@zs-hospital.sh.cn; xu.huixiong@zs-hospital.sh.cn;


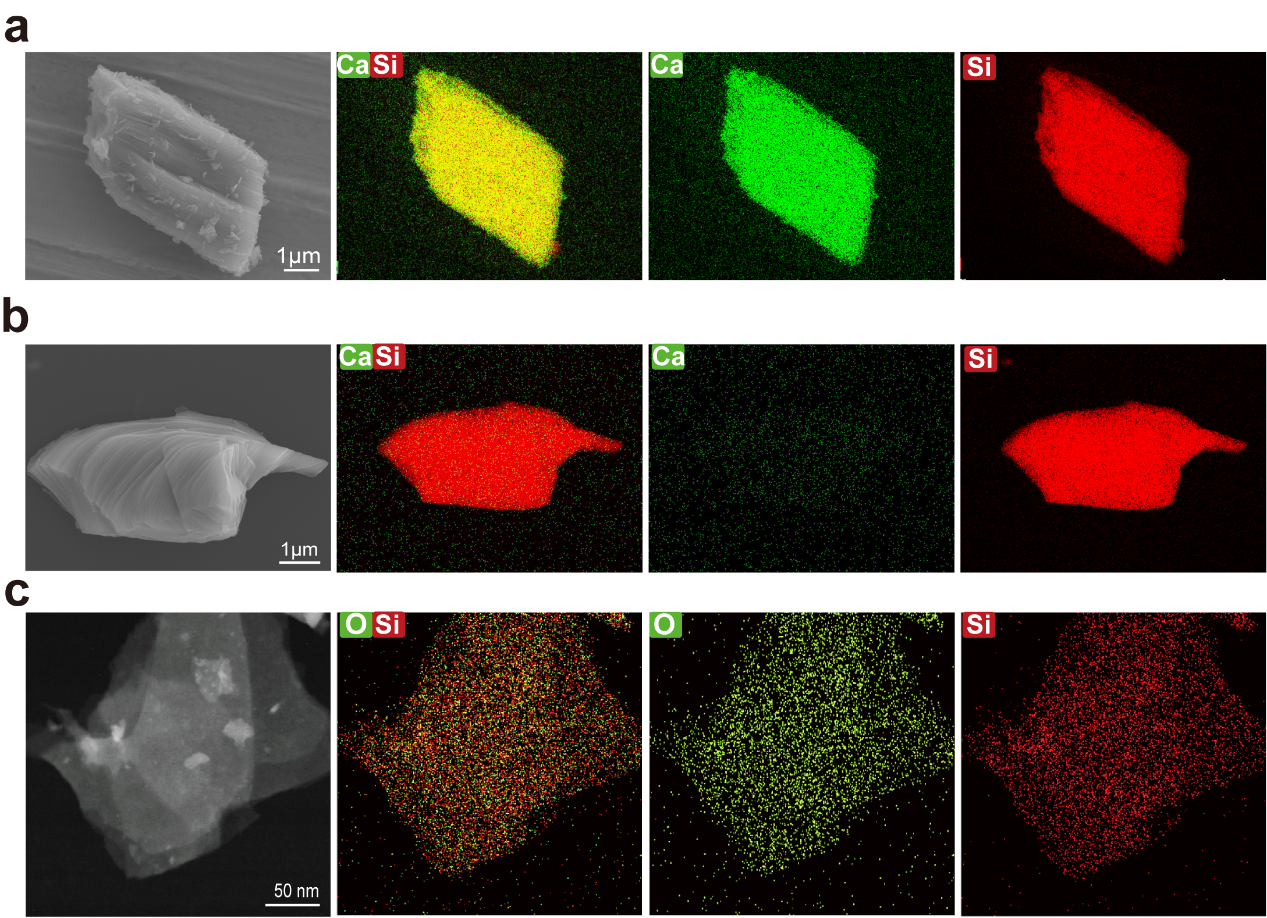


**Fig. S1** a) Corresponding elemental-mapping images of CaSi_2_ (Ca and Si elements). b) of SiH with large size before sonication (Ca and Si elements). c) of SiH nanosheets (Si and O elements).

**
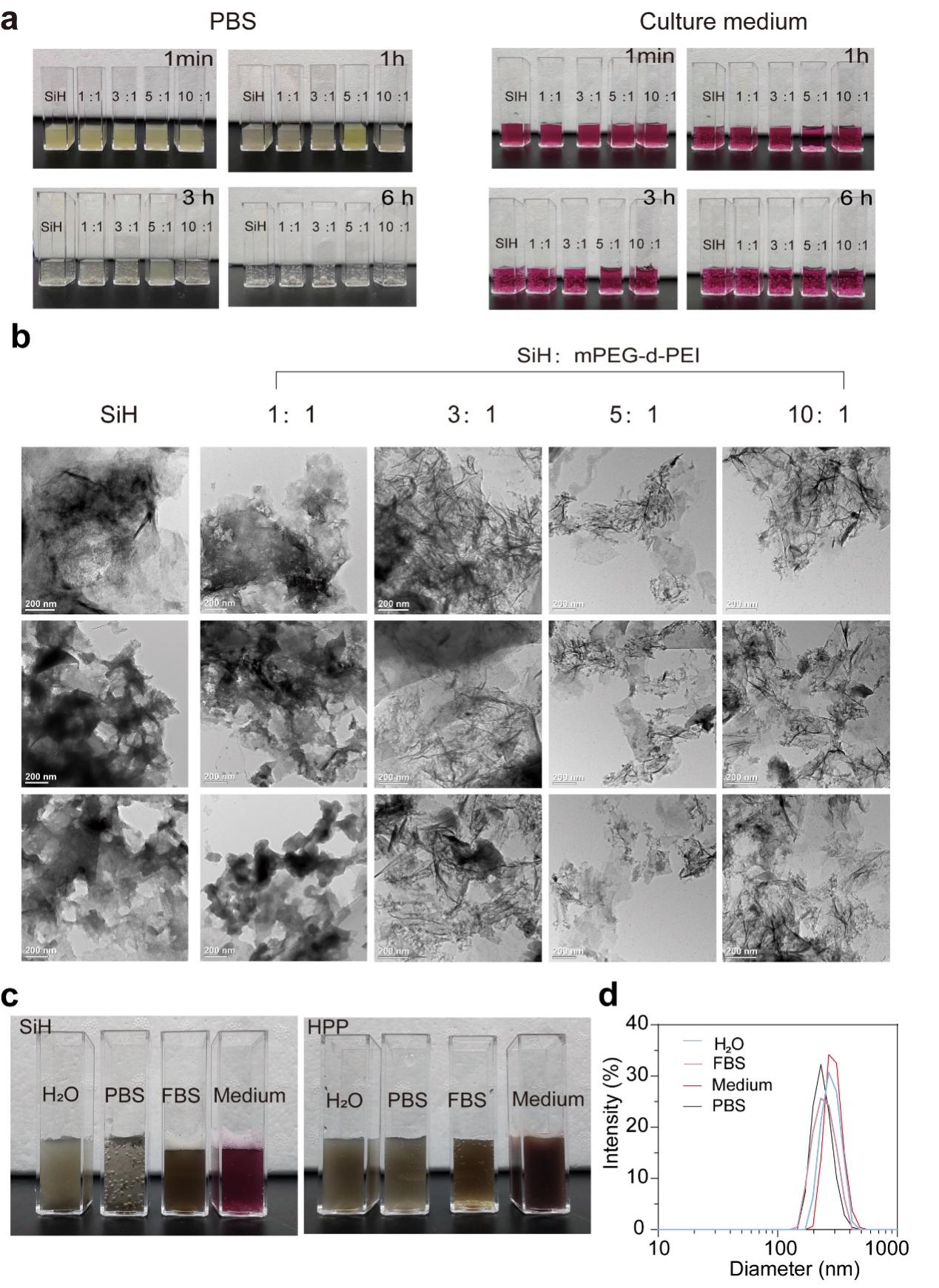
**

**Fig. S2** a) The digital photographs of HPP (SiH and mPEG-d-PEI with mass ratios of 1:0, 1:1, 3:1, 5:1, and 10:1) dispersed in ethanol and reacted with PBS, and culture medium solutions for 1 min,1 h, 3 h, 6 h at room temperature. b) SiH and mPEG-d-PEI were combined in different mass ratios (1:1, 3:1, 5:1, and 10:1). The TEM image showed the product after reacting with PBS for 6 h (n = 3 independent samples). c) The digital photographs of SiH before and after modification (SiH: mPEG-d-PEI at 5:1) dispersed in different solutions (water, PBS, FBS, Medium) for 1 h. d) Hydrodynamic diameters of SiH after mPEG-d-PEI modification in various physiological solutions including water, PBS, FBS and culture medium.


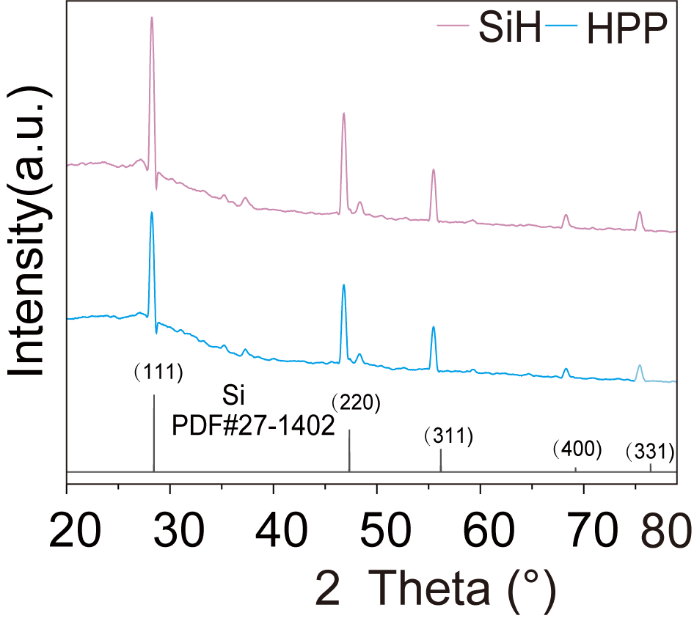


**Fig. S3** X-ray diffraction (XRD) patterns of SiH and HPP nanosheets.


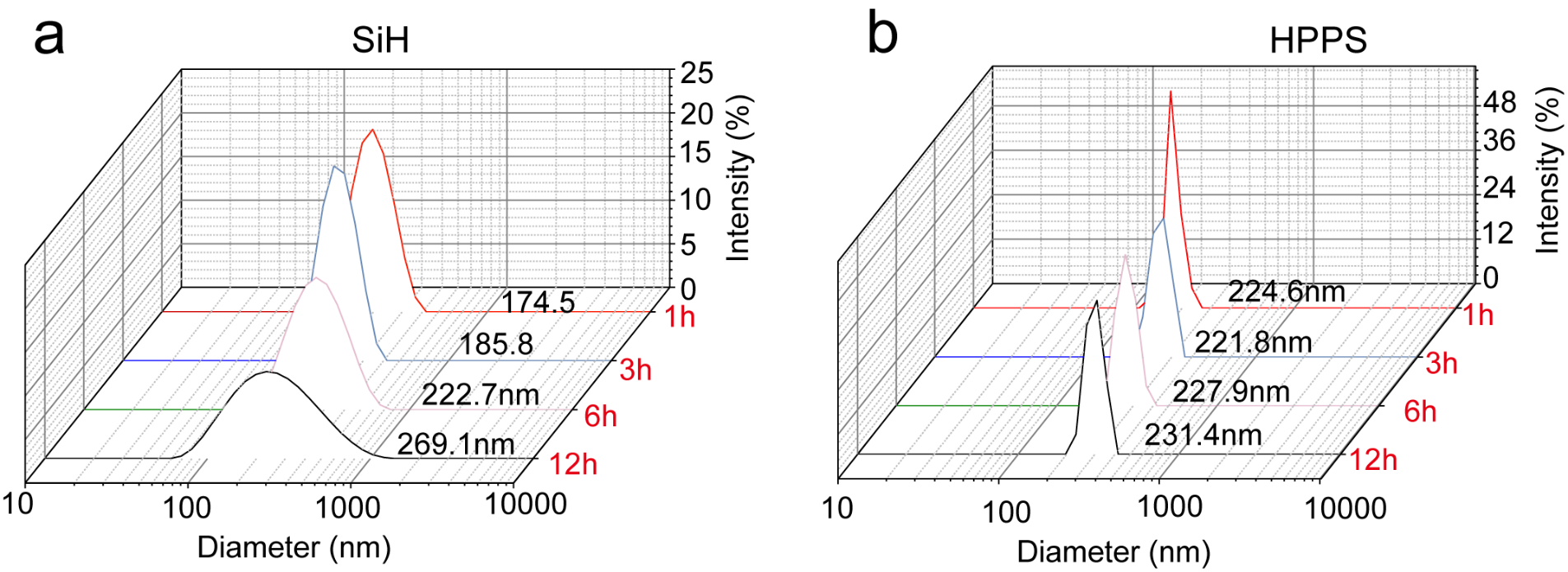


**Fig. S4** Particle size distribution and stability of SiH (a) and HPPS (b) in PBS at various time points (1 h, 3 h, 6 h, and 12 h).


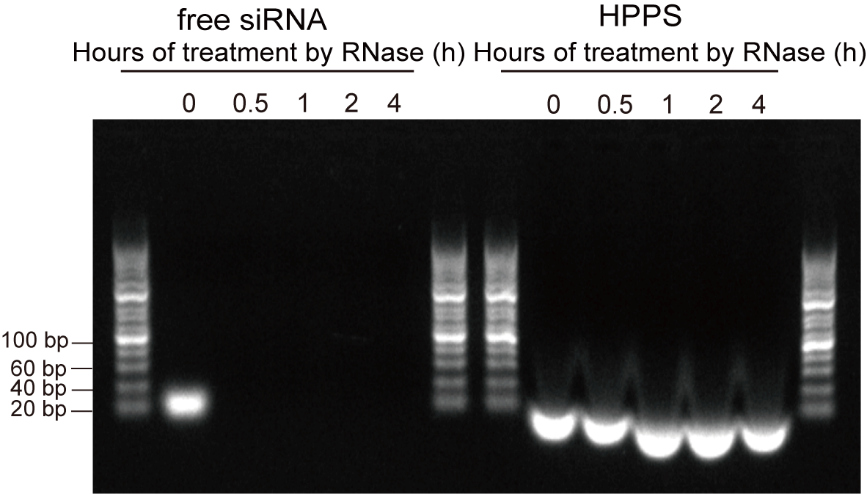


**Fig. S5** Stability of naked siRNA and HPPS in RNase. siRNA was extracted by adding SDS (n = 3 independent samples).


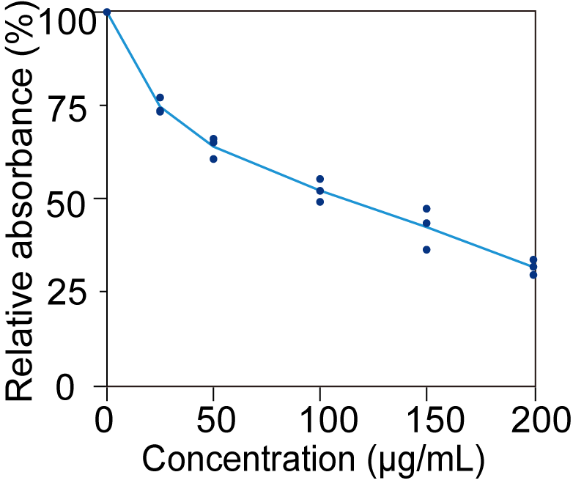


**Fig. S6** Evaluations of ABTS+ scavenging capability of HPPS (n = 3 independent samples).


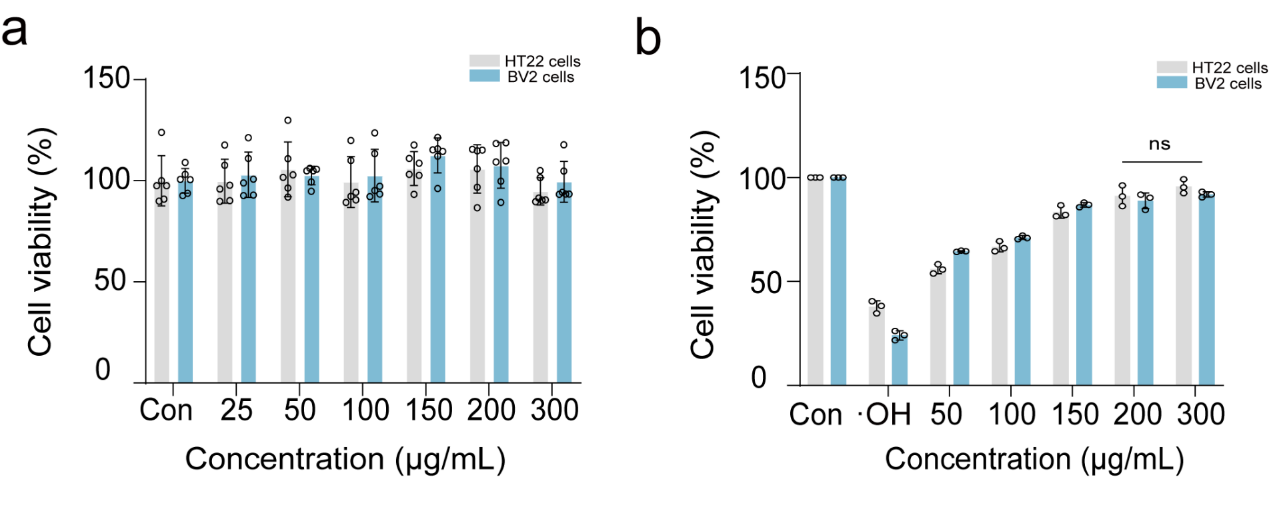


**Fig. S7** Viability of HT22 and BV2 cells co-incubated with a) various concentrations of HPPS for 12 h (0, 25, 50, 100, 200 and 300 μg/ mL) (n = 5 biologically independent experiments). b) Fenton reagent and different concentrations of HPPS for 12 h (0, 25, 50, 100, 200 and 300 μg/ mL) (n=3 biologically independent experiments). Data are presented as Mean ± SD. *****P* < 0.0001, ****P* < 0.001, ***P* < 0.01, **P* < 0.05, ns: no significance.


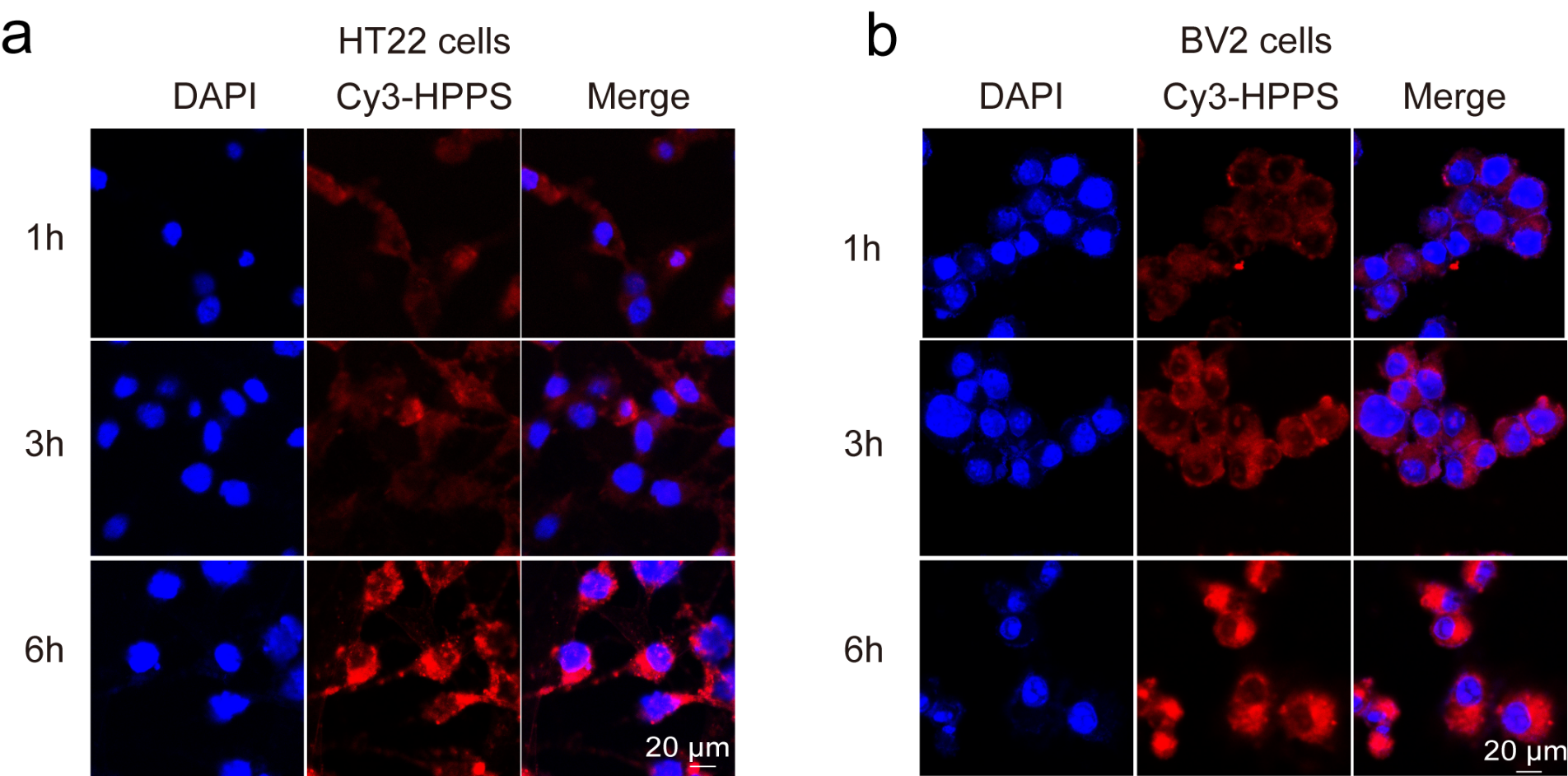


**Fig. S8** a, b) CLSM images of Cy3-labeled HPPS co-incubated with HT22 and BV2 cells for 1, 3, 6 h (n = 3 biologically independent experiments).


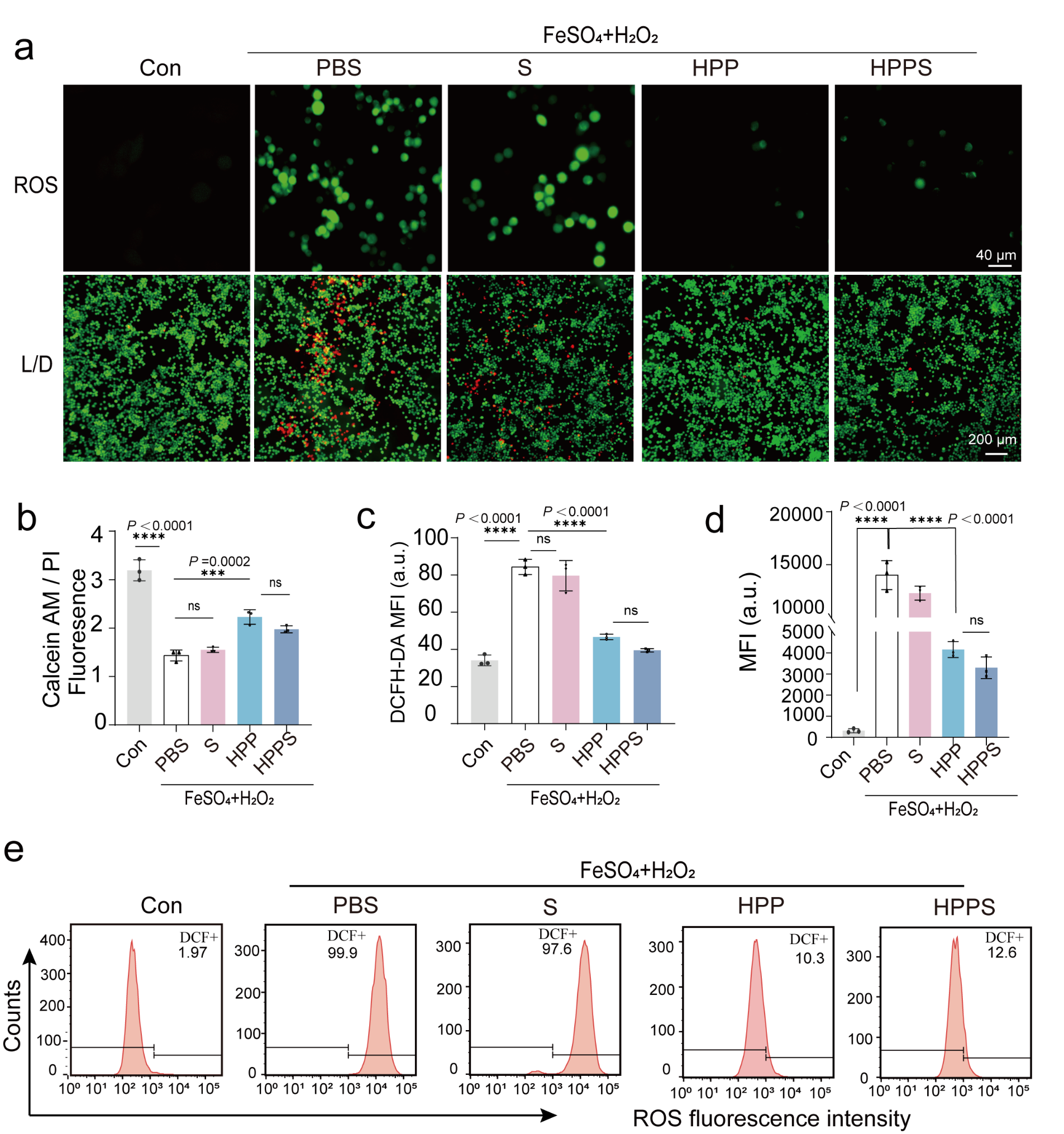


**Fig. S9** a) CLSM images of BV2 cells stained with DCFH-DA and Calcein-AM/PI (L/D represents Live/Dead) with Fenton regen and various treatments (Con, PBS, siRNA, HPP, HPPS). HPP = 200 μg/mL, siRNA=25nM. Incubation time =12 h. A representative image of three replicates from each group is shown (n=3 biologically independent experiments). b) Corresponding fluorescence intensity of calcein-AM / propidium iodide (PI) of BV2 cells with Fenton regen and various treatments (Con, PBS, siRNA, HPP, HPPS) (n = 3 biologically independent experiments) c) ROS fluorescence intensity of CLSM images of BV2 cells with Fenton regen and various treatments (Con, PBS, siRNA, HPP, HPPS) (n=3 biologically independent experiments). (d) Flow cytometry (FCM) analysis of BV2 ROS intensity corresponding fluorescence intensities with Fenton regen and various treatments (Con, PBS, siRNA, HPP, HPPS) (n=3 biologically independent experiments). (e) Typical flow cytometric of ROS in BV2 cells with Fenton regen and various treatments (Con, PBS, siRNA, HPP, HPPS) (n = 3 biologically independent experiments). Data are presented as Mean ± SD. *****P* < 0.0001, ****P* < 0.001, ***P* < 0.01, **P* < 0.05, ns: no significance.


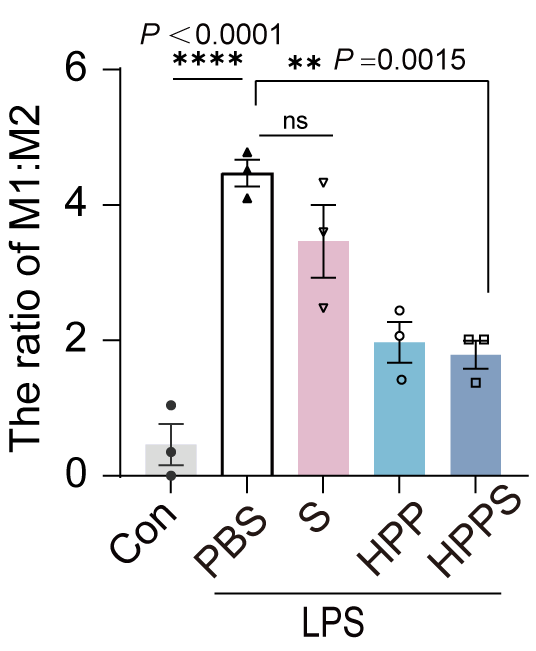


**Fig. S10** Histogram showed that HPPS decrease M1/M2 polarization ratio under LPS (Con, PBS, siRNA, HPP, HPPS) (n = 3 biologically independent experiments). Data are presented as Mean ± SD. *****P* < 0.0001, ****P* < 0.001, ***P* < 0.01, **P* < 0.05, ns: no significance.


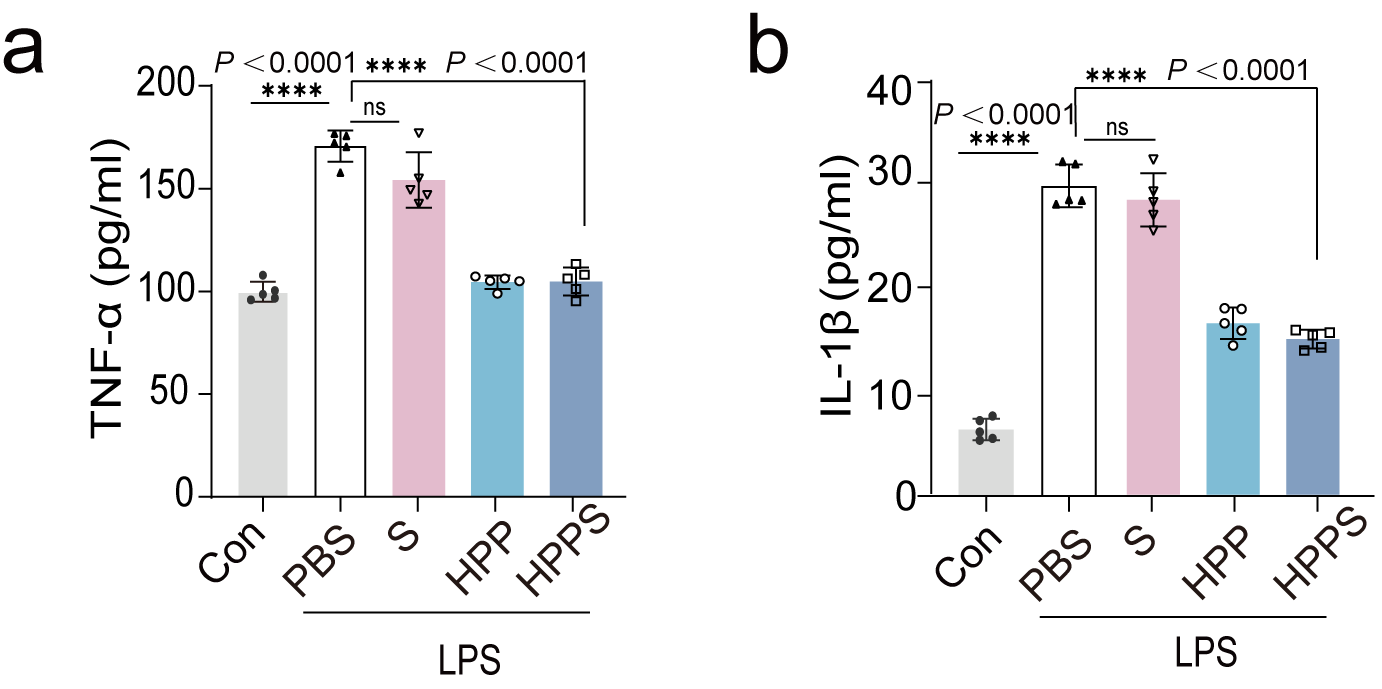


**Fig. S11** a, b) Inflammatory cytokine TNF-α, IL-1β secretion levels of BV2 Cells under LPS and different Treatments (Con, PBS, siRNA, HPP, HPPS) (n = 6 biologically independent experiments). Data are presented as Mean ± SD. *****P* < 0.0001, ****P* < 0.001, ***P* < 0.01, **P* < 0.05, ns: no significance.


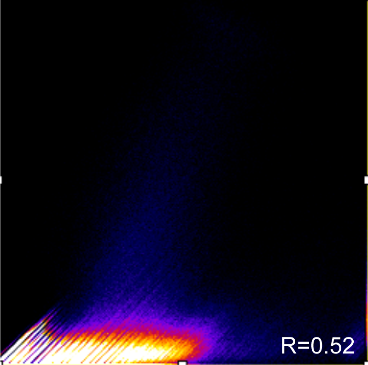


**Fig. S12** Confocal imaging study of HT22 cells co-staining with lysotracker Green and Cy3-labled HPPS. R is the correlation coefficient.


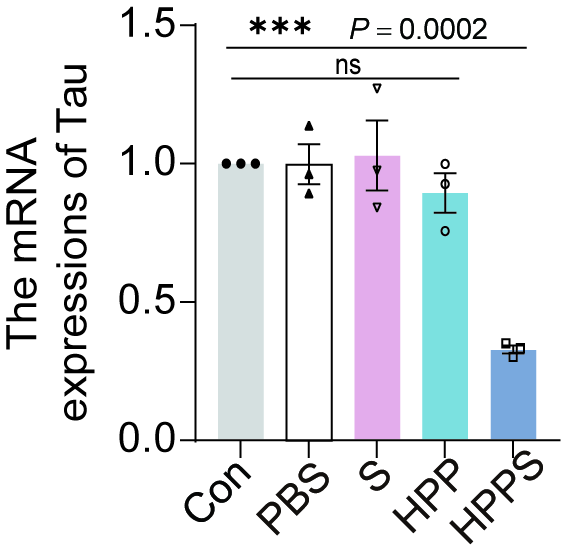


**Fig. S13** qRT-PCR analysis of tau mRNA in HT22 cells of different groups (Con, PBS, S, HPP, HPPS; n = 3 biologically independent experiments). Data are presented as Mean ± SD. *****P* < 0.0001, ****P* < 0.001, ***P* < 0.01, **P* < 0.05, ns: no significance.


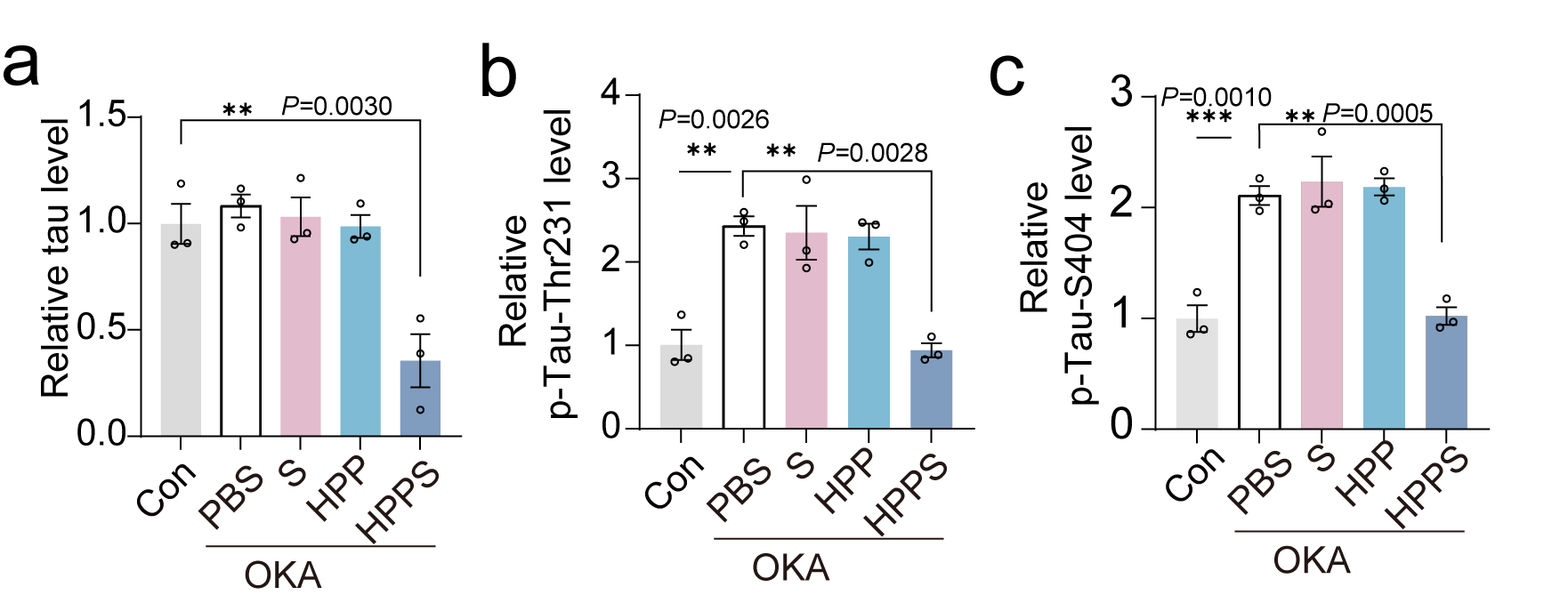


**Fig. S14** a, b, c) Corresponding quantitative analysis of western blot analysis for tau, p-tau-Thr231, p-tau-S404 of OKA-stimulated HT22 cells with various treatments (Con, PBS, S, HPP, HPPS) (n = 3 biologically independent experiments). Data are presented as Mean ± SD. *****P* < 0.0001, ****P* < 0.001, ***P* < 0.01, **P* < 0.05, ns: no significance.


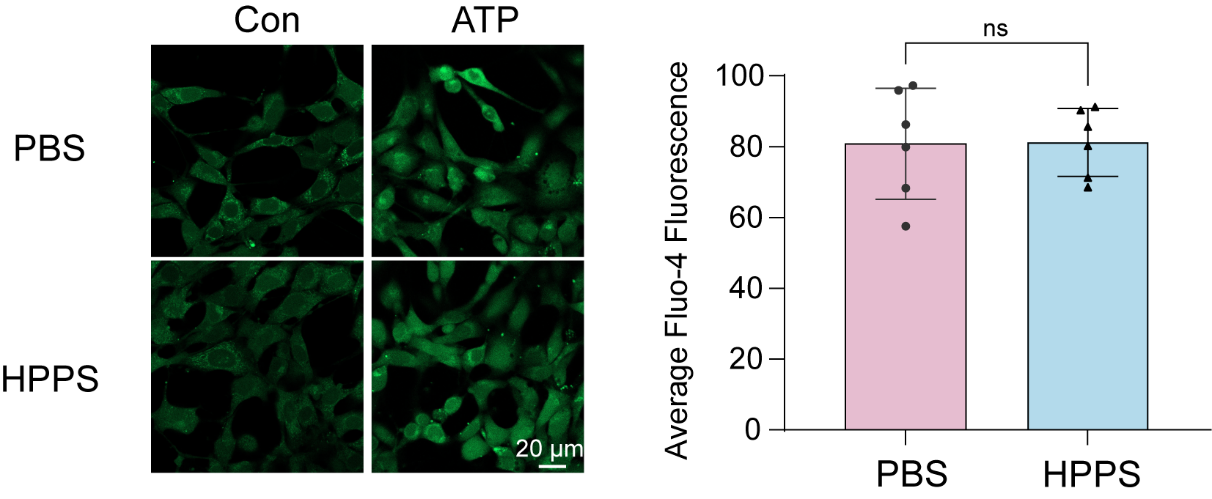


**Fig. S15** Effects of HPPS treatment on calcium influx in HT22 cells. Representative fluorescence images of HT22 cells stained with Fluo-4 AM and quantitative analysis of average Fluo-4 fluorescence intensity (n = 6 biologically independent experiments).

Data are presented as Mean ± SD. *****P* < 0.0001, ****P* < 0.001, ***P* < 0.01, **P* < 0.05, ns: no significance.


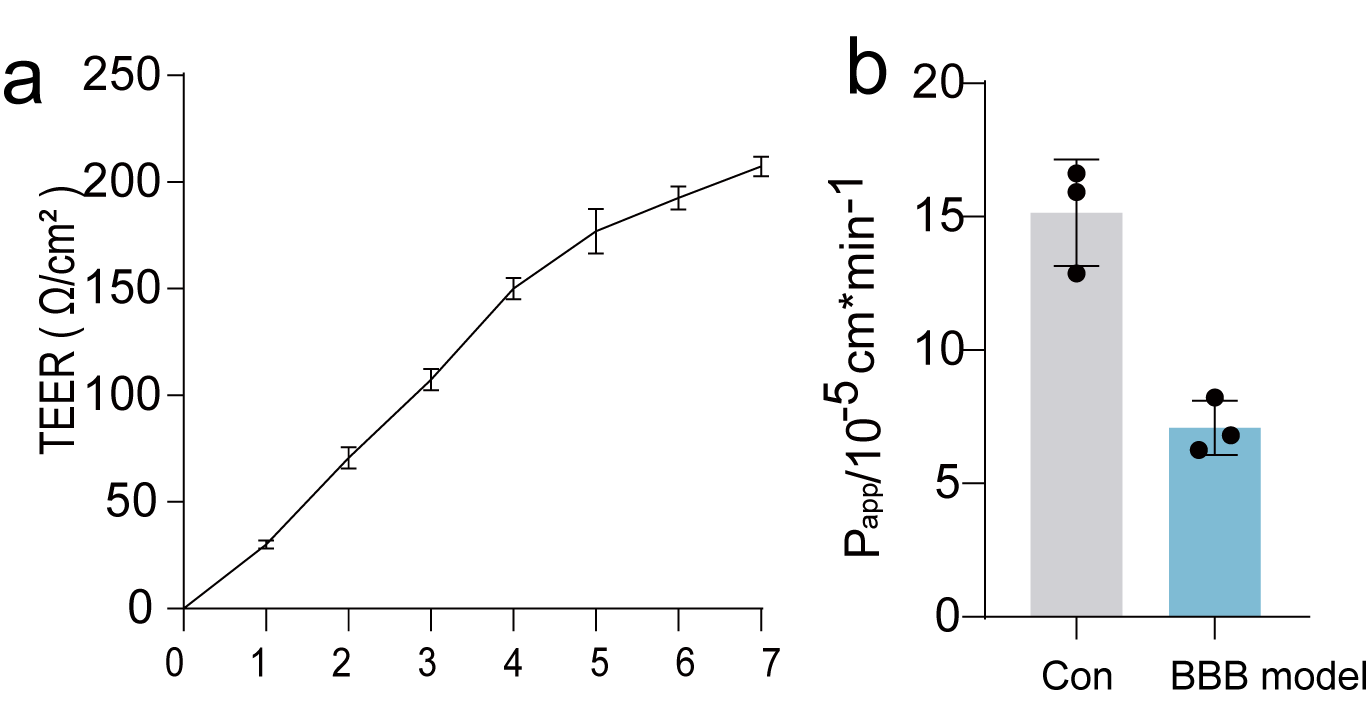


**Fig. S16.** a) TEER values over seven days during BBB establishment. b) Comparison of Na-Flu permeability between control and model groups after successful BBB construction (n = 3 biologically independent experiments).


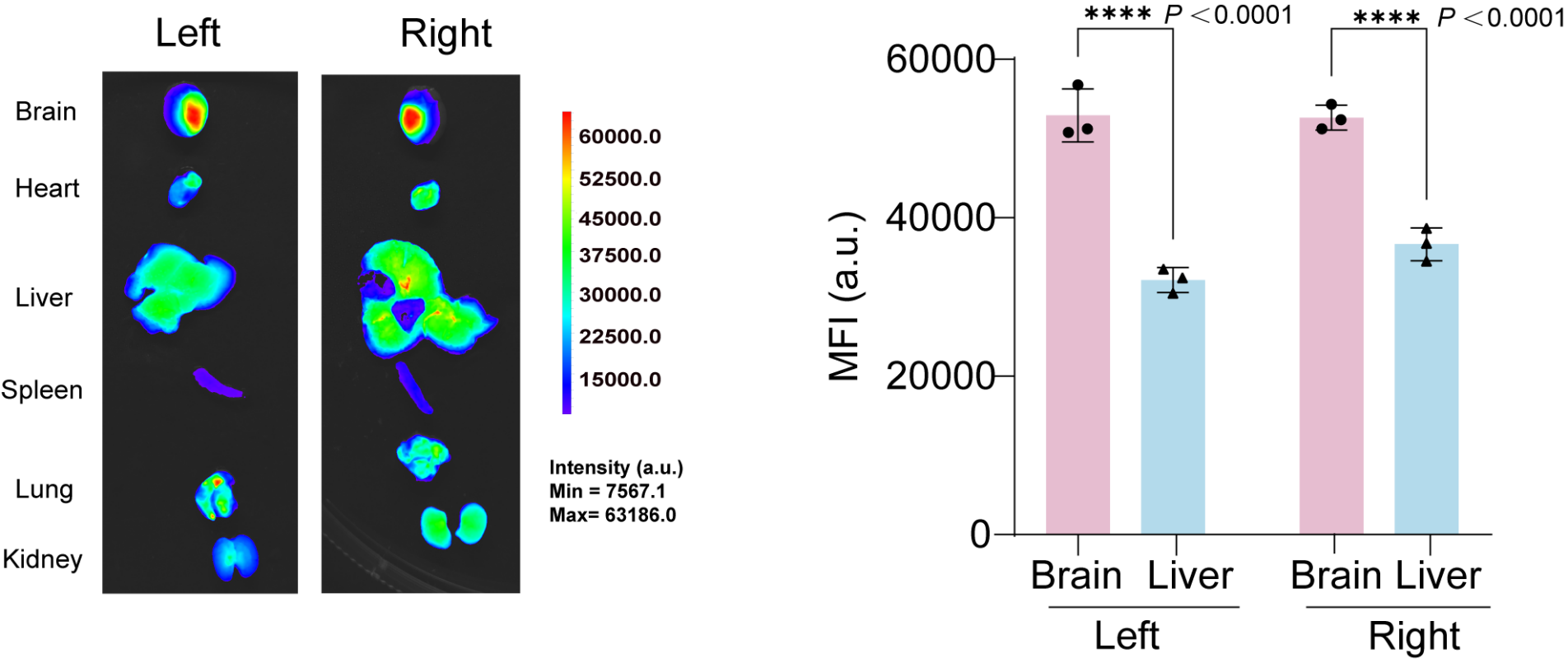


**Fig. S17** Ex vivo fluorescence imaging and quantification of HPPS distribution in various organs, including the brain, heart, liver, spleen, lung, and kidney, 1 h post-FUS irradiation (n = 3 biologically independent experiments). Data are presented as Mean ± SD. *****P* < 0.0001, ****P* < 0.001, ***P* < 0.01, **P* < 0.05, ns: no significance.


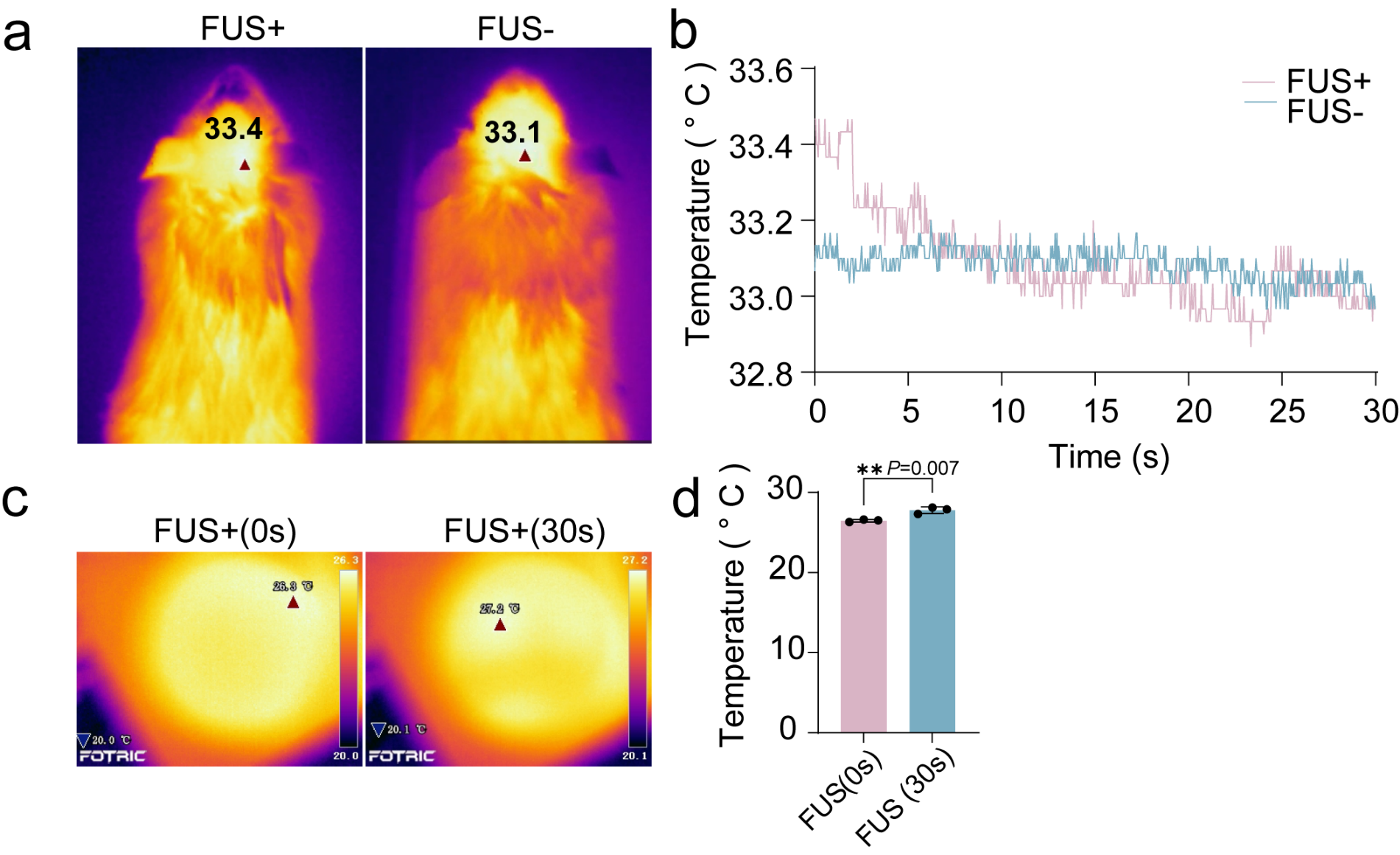


**Fig. S18** Evaluation of the thermal effects during ultrasound treatment. a) Infrared thermal images showing the temperature of the head skin specifically at the ultrasound-treated area at the end of ultrasound exposure (FUS+), compared to the untreated control area (FUS-) (n = 3 biologically independent experiments). b) Continuous monitoring of head skin temperature for 30 s after ultrasound treatment. c) In vitro measurement of the temperature increase in HT22 cells exposed to ultrasound. d) Quantification of temperature increase in HT22 cells (n = 3 biologically independent experiments). Data are presented as Mean ± SD. *****P* < 0.0001, ****P* < 0.001, ***P* < 0.01, **P* < 0.05, ns: no significance.


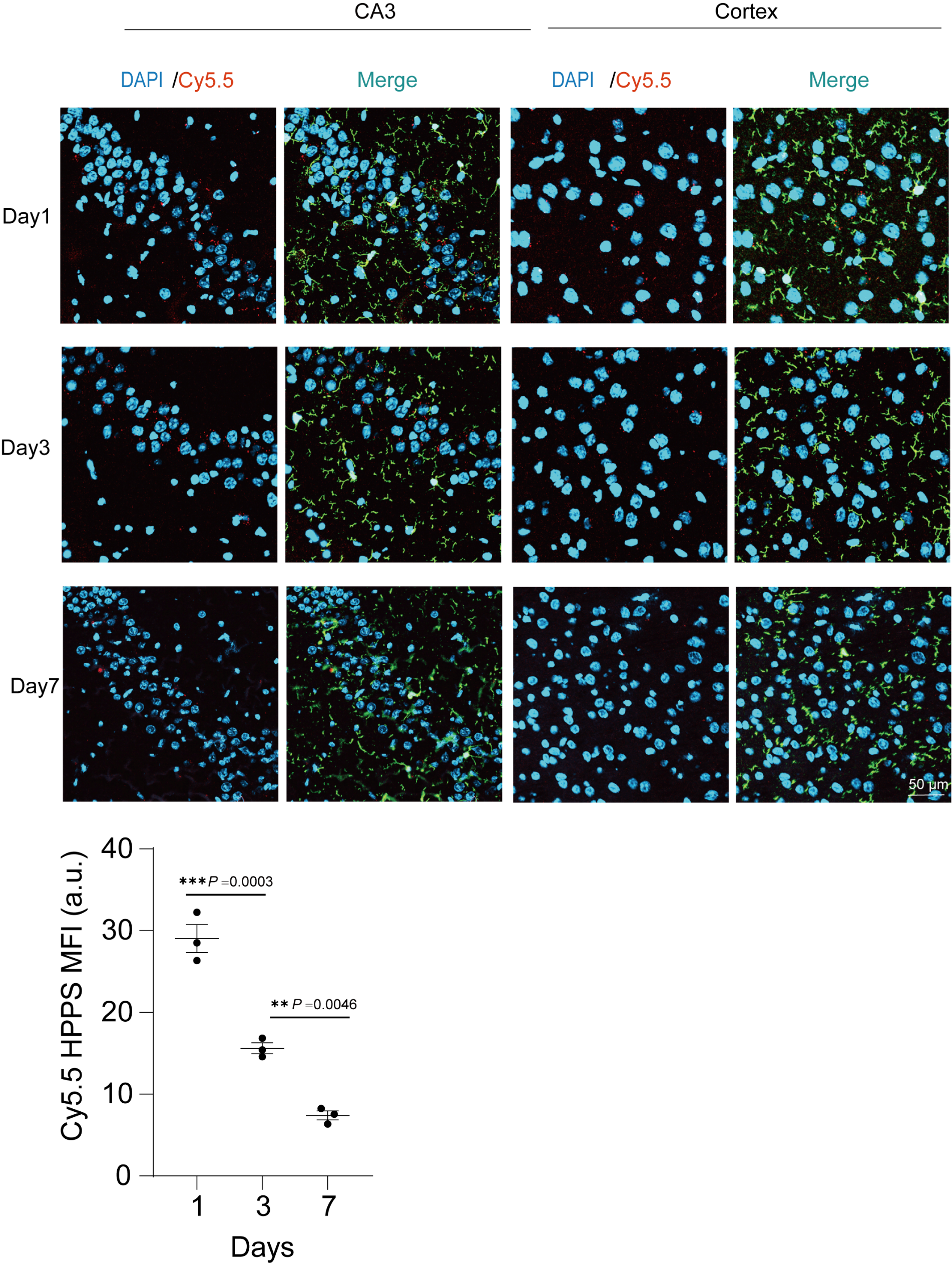


**Fig.S19** Representative immunofluorescence images of cy5.5-HPPS in the hippocampus and cortex (1,3,7 d) (n = 3 biologically independent experiments). Data are presented as Mean ± SD. *****P* < 0.0001, ****P* < 0.001, ***P* < 0.01, **P* < 0.05, ns: no significance.


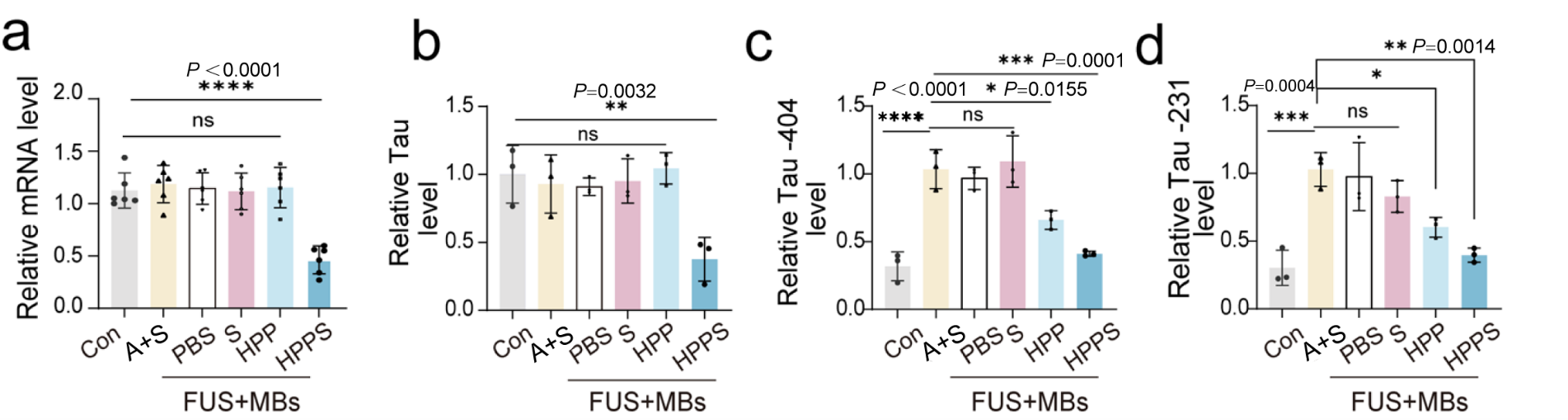


**Fig.S20** a) Expression levels of tau protein mRNA at 48 h post-injection (Con, A+S, PBS, S, HPP, HPPS) (n = 6 biologically independent experiments). b, c, d) Corresponding quantitative analysis of western blot analysis of tau protein, and phosphorylated tau protein in hippocampal tissue at 48 h post-injection (Con, A+S, PBS, S, HPP, HPPS) (n = 3 biologically independent experiments). Data are presented as Mean ± SD. *****P* < 0.0001, ****P* < 0.001, ***P* < 0.01, **P* < 0.05, ns: no significance.


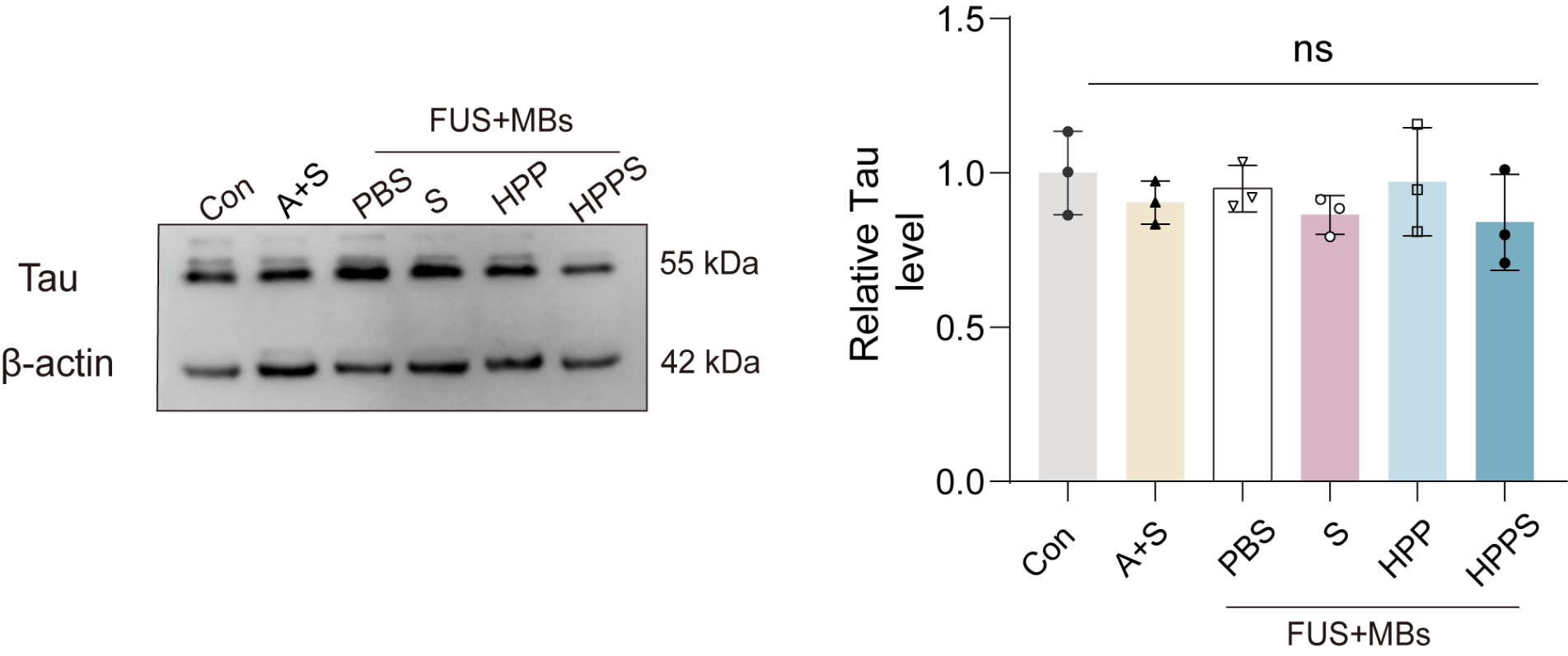


**Fig.S21** Western blot analysis and quantification of tau protein expression in the hippocampus on the seventh day post-surgery (n = 3 biologically independent experiments). Data are presented as Mean ± SD. *****P* < 0.0001, ****P* < 0.001, ***P* < 0.01, **P* < 0.05, ns: no significance.


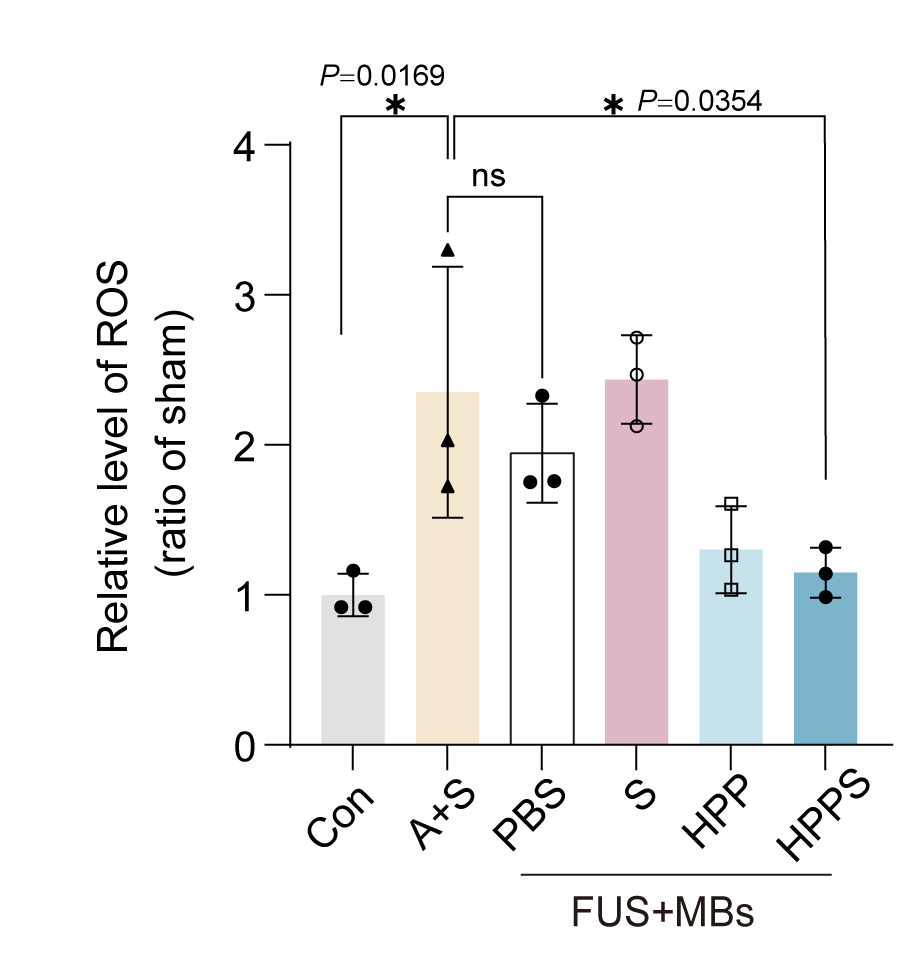


**Fig.S22** Relative level of hippocampal ROS in group Con, A+S, PBS, S, HPP, HPPS (n = 3 biologically independent experiments). Data are presented as Mean ± SD. *****P* < 0.0001, ****P* < 0.001, ***P* < 0.01, **P* < 0.05, ns: no significance.


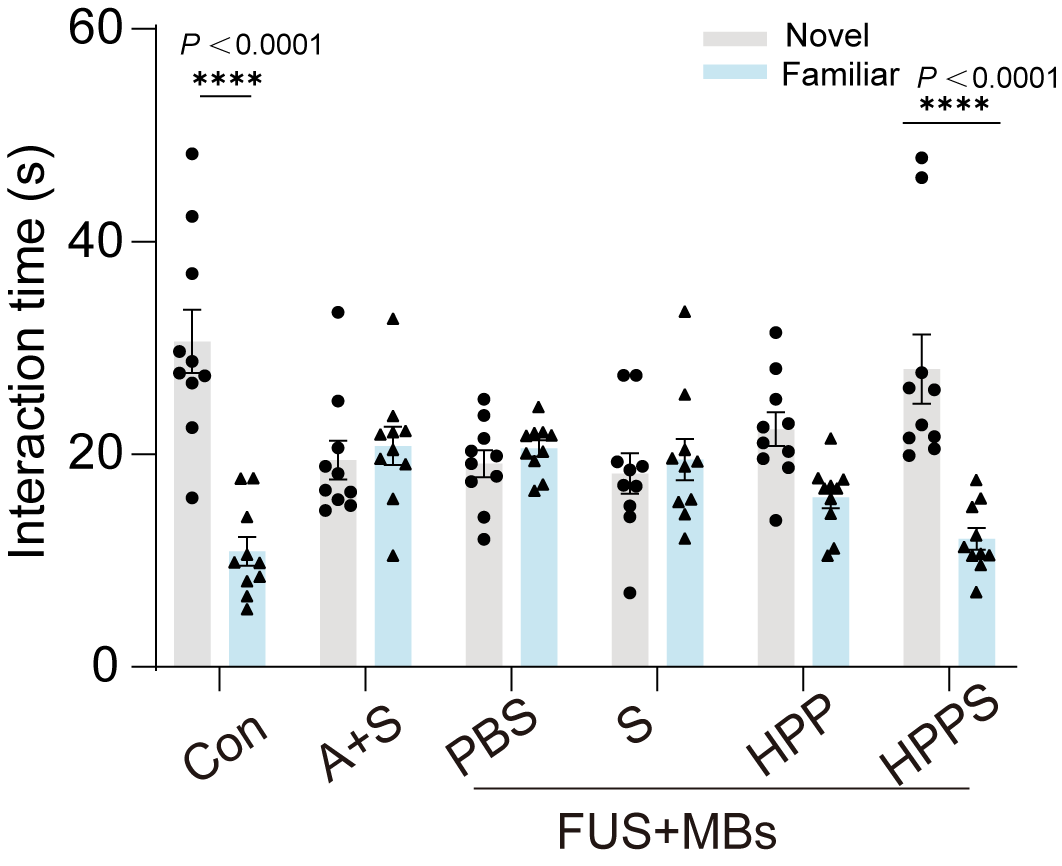


**Fig.S23** The time spent interacting with novel and familiar object in the NOR (Con, A+S, PBS, S, HPP, HPPS) (n=10 biologically independent experiments). Data are presented as Mean ± SD. *****P* < 0.0001, ****P* < 0.001, ***P* < 0.01, **P* < 0.05, ns: no significance.


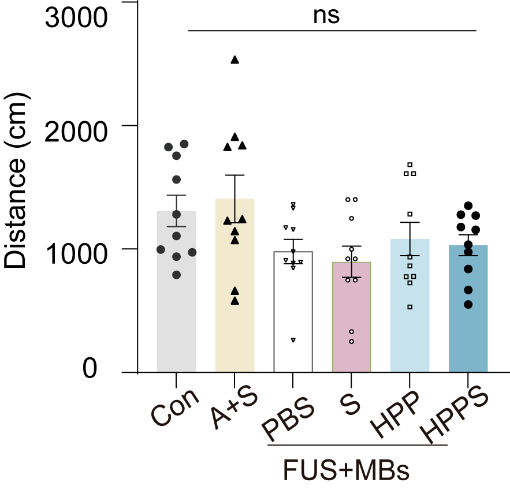


**Fig.S24** Total distance in the open field test (Con, A+S, PBS, S, HPP, HPPS) (n=10 biologically independent experiments). Data are presented as Mean ± SD. *****P* < 0.0001, ****P* < 0.001, ***P* < 0.01, **P* < 0.05, ns: no significance.


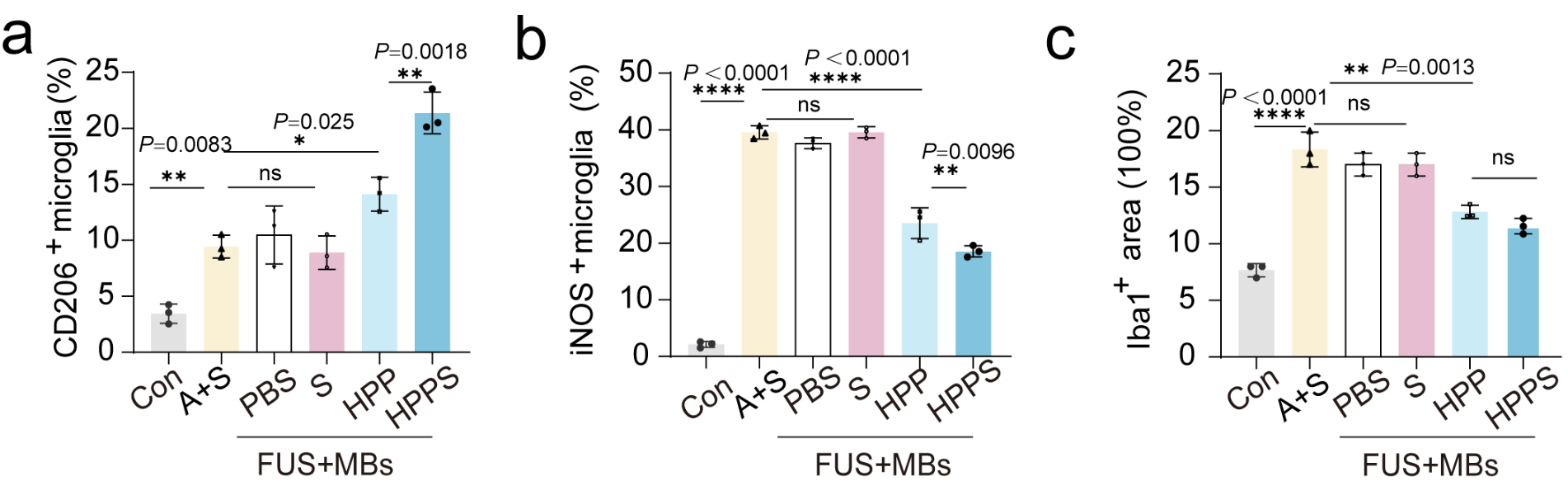


**Fig.S25** a, b) Quantification of the percentage of lba1^+^/CD206^+^ and lba1^+^/inos cells cells among lba1^+^ cells in the CA1, CA3, and DG regions of different groups (Con, A+S, PBS, S, HPP, HPPS). c) Quantification of lba1^+^ area in hippocampus (Con, A+S, PBS, S, HPP, HPPS) (n=3 biologically independent experiments). Data are presented as Mean ± SD. *****P* < 0.0001, ****P* < 0.001, ***P* < 0.01, **P* < 0.05, ns: no significance.


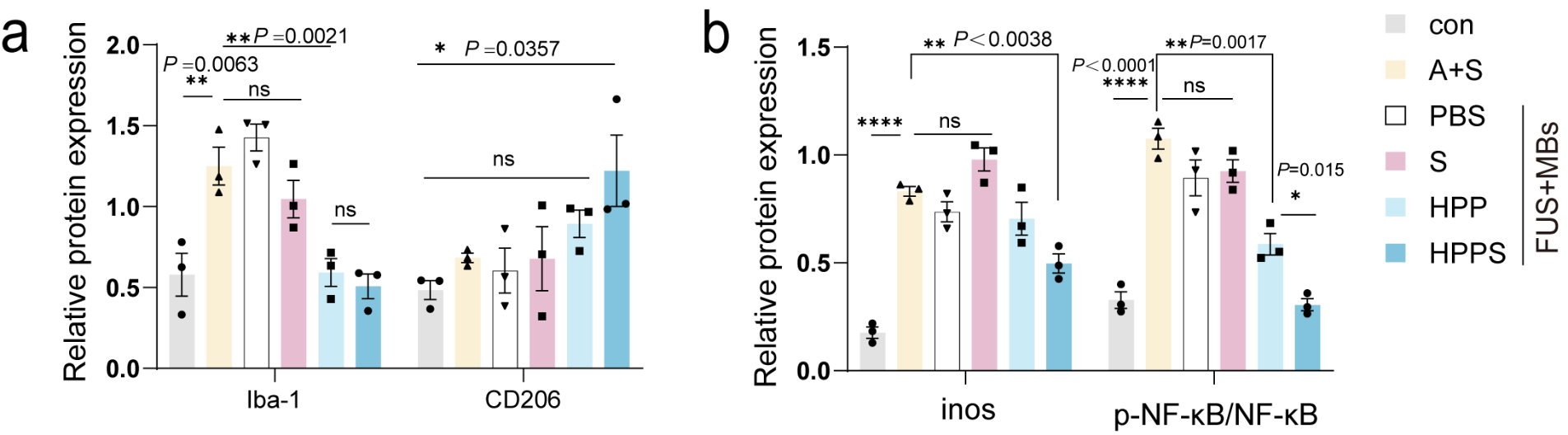


**Fig.S****26** a,b) Corresponding quantitative analysis of western blot analysis of lba-1, CD206, inos, NF-κB p65, NF-κB p- p65 (Con, A+S, PBS, S, HPP, HPPS) (n=3 biologically independent experiments). Data are presented as Mean ± SD. *****P* < 0.0001, ****P* < 0.001, ***P* < 0.01, **P* < 0.05, ns: no significance.


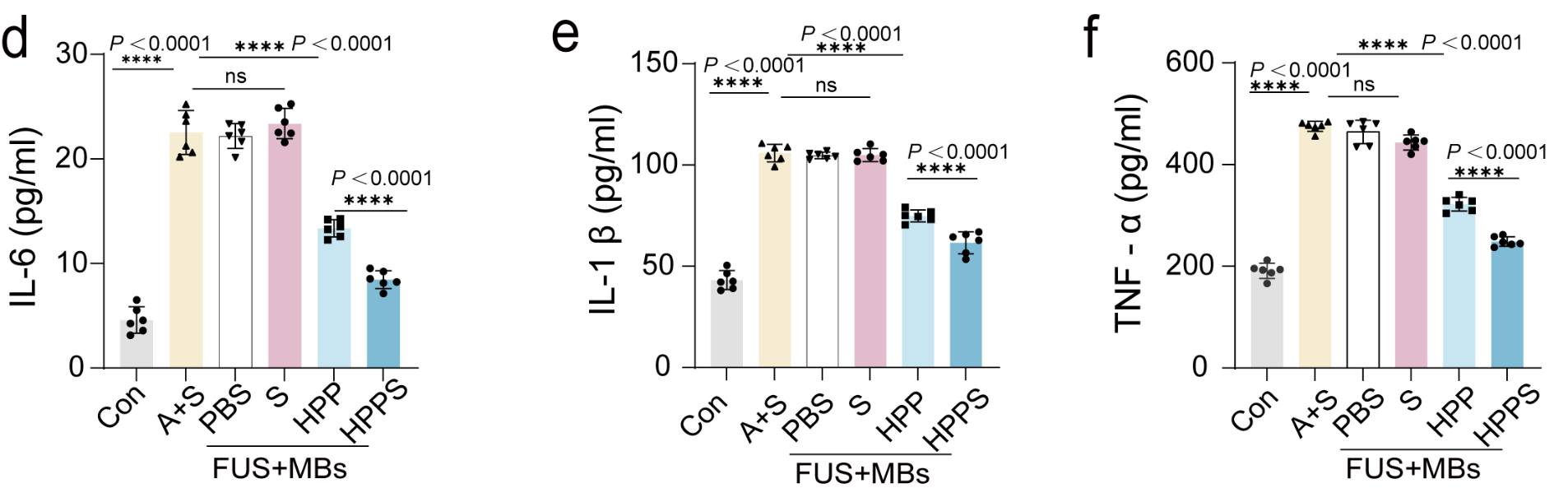


**Fig.S27** a, b, c) ELISA measurements of IL-6, IL-1β, and TNF-α levels in hippocampal tissue of different groups (Con, A+S, PBS, S, HPP, HPPS) (n = 6 biologically independent experiments). Data are presented as Mean ± SD. *****P* < 0.0001, ****P* < 0.001, ***P* < 0.01, **P* < 0.05, ns: no significance.


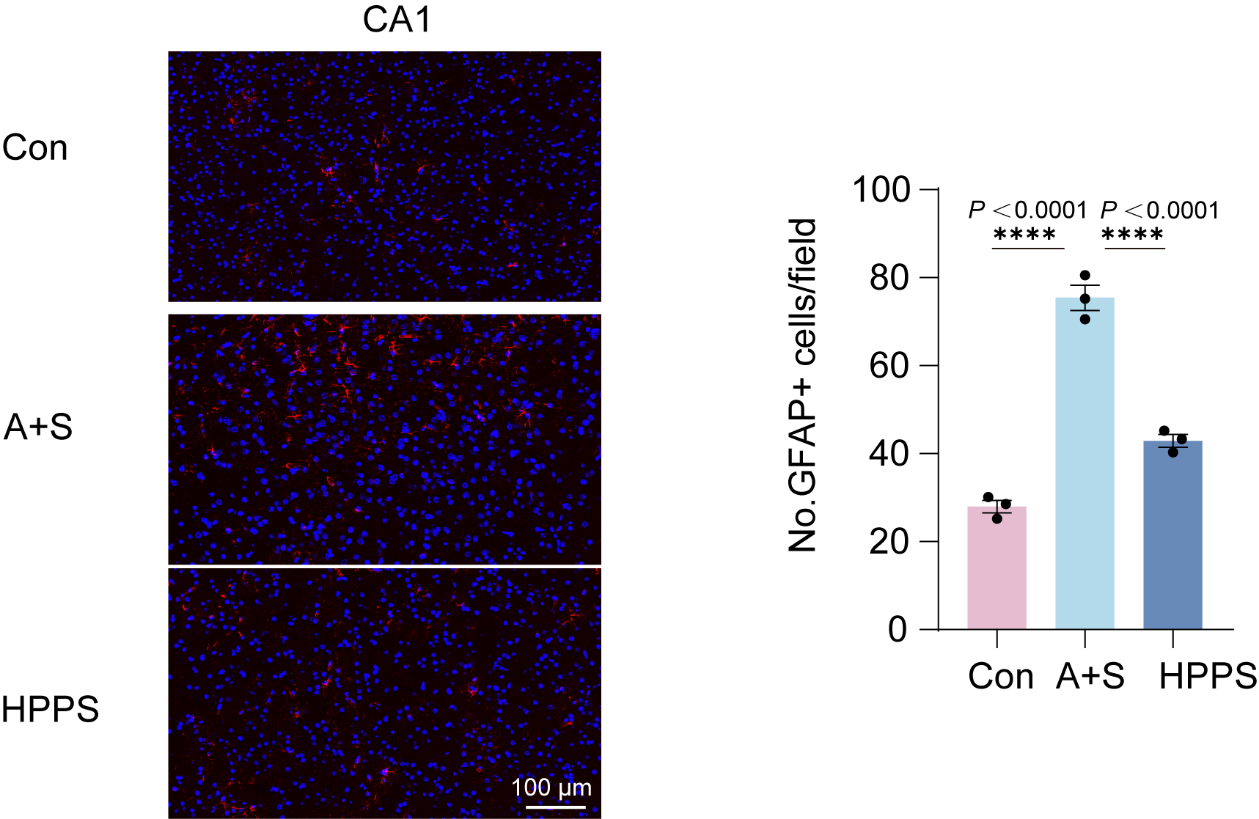


**Fig.S28** Immunofluorescence staining and quantification of GFAP+ astrocytes in the hippocampal CA1 region (n = 3 biologically independent experiments). Data are presented as Mean ± SD. *****P* < 0.0001, ****P* < 0.001, ***P* < 0.01, **P* < 0.05, ns: no significance.


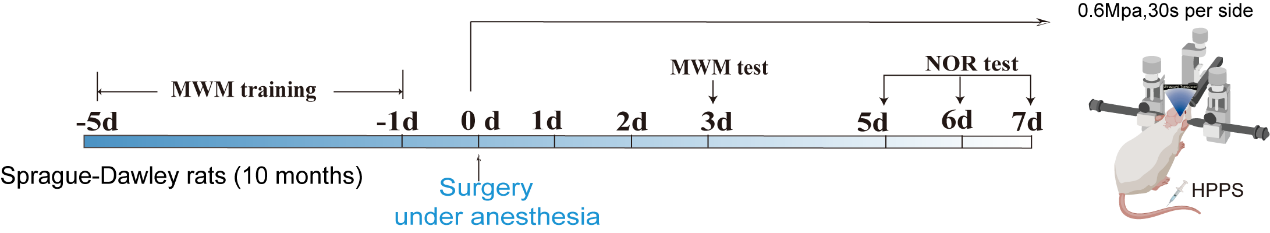


**Fig.S29** Timeline diagram of behavioral testing in Sprague-Dawley rats following partial hepatectomy.


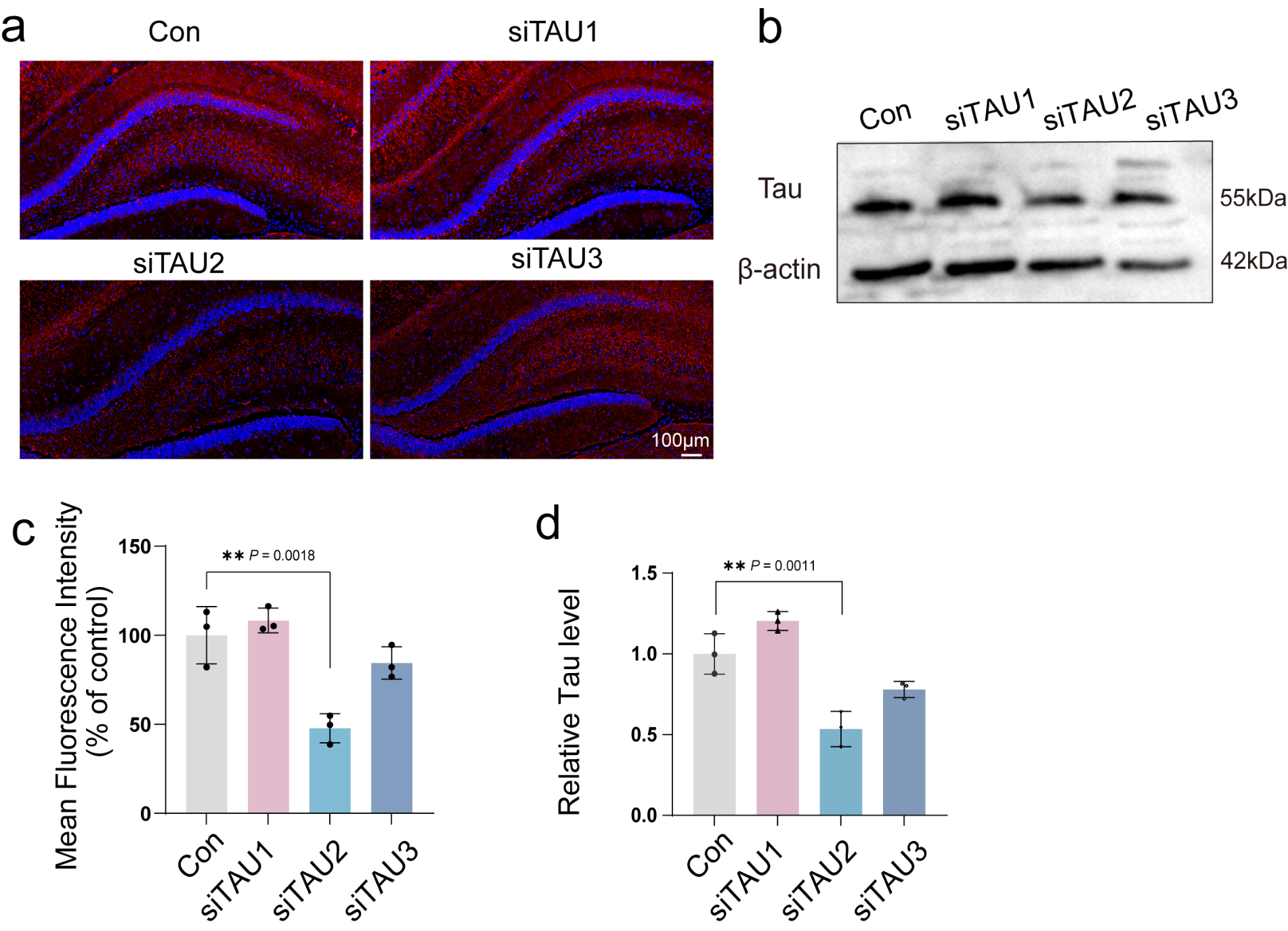


**Fig.S30** Validation of tau knockdown efficiency using three tau-siRNA sequences (siTAU1, siTAU2, siTAU3) in rat. a) Representative immunofluorescence images showing tau expression levels in the hippocampus across different groups (Con, siTAU1, siTAU2, siTAU3), with tau (red) and nuclei (blue) staining. b) Western blot analysis of tau protein expression in the hippocampus. (c) Quantification of mean fluorescence intensity from immunofluorescence images, expressed as a percentage of the control group. (d) Quantification of relative tau protein levels from Western blot analysis (n = 3 biologically independent experiments). Data are presented as Mean ± SD. *****P* < 0.0001, ****P* < 0.001, ***P* < 0.01, **P* < 0.05, ns: no significance.

.


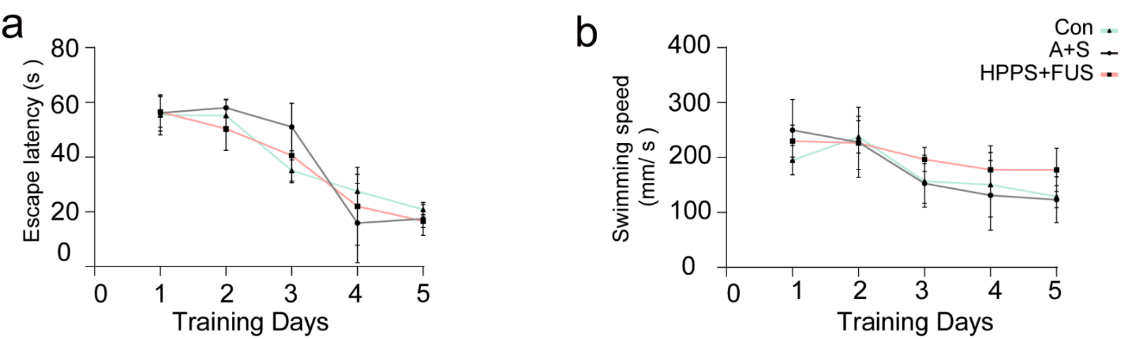


**Fig.S31** a, b）The mean escape latency and swimming speed in the hidden platform test in the 5 d of training of different groups (Con, A+S, HPPS+FUS) (n = 6 biologically independent experiments).
